# Fusion of a Novel Native Signal Peptide Enhanced the Secretion and Solubility of Bioactive Human Interferon Gamma Glycoproteins in *Nicotiana benthamiana* Using the *Bamboo Mosaic Virus*-Based Expression System

**DOI:** 10.3389/fpls.2020.594758

**Published:** 2020-11-12

**Authors:** Min-Chao Jiang, Chung-Chi Hu, Wei-Li Hsu, Tsui-Ling Hsu, Na-Sheng Lin, Yau-Heiu Hsu

**Affiliations:** ^1^Ph.D. Program in Microbial Genomics, National Chung Hsing University and Academia Sinica, Taichung, Taiwan; ^2^Graduate Institute of Biotechnology, National Chung Hsing University, Taichung, Taiwan; ^3^Advanced Plant Biotechnology Center, National Chung Hsing University, Taichung, Taiwan; ^4^Graduate Institute of Microbiology and Public Health, College of Veterinary Medicine, National Chung Hsing University, Taichung, Taiwan; ^5^Genomics Research Center, Academia Sinica, Taipei, Taiwan; ^6^Institute of Plant and Microbial Biology, Academia Sinica, Taipei, Taiwan

**Keywords:** interferon gamma, signal peptides, anti-virus activity, plant viral vector, glycosylation, *Bamboo mosaic virus*

## Abstract

Plant viruses may serve as expression vectors for the efficient production of pharmaceutical proteins in plants. However, the downstream processing and post-translational modifications of the target proteins remain the major challenges. We have previously developed an expression system derived from *Bamboo mosaic virus* (BaMV), designated pKB19, and demonstrated its applicability for the production of human mature interferon gamma (mIFNγ) in *Nicotiana benthamiana*. In this study, we aimed to enhance the yields of soluble and secreted mIFNγ through the incorporation of various plant-derived signal peptides. Furthermore, we analyzed the glycosylation patterns and the biological activity of the mIFNγ expressed by the improved pKB19 expression system in *N. benthamiana*. The results revealed that the fusion of a native *N. benthamiana* extensin secretory signal (SS^Ext^) to the N-terminal of mIFNγ (designated SS^Ext^ mIFNγ) led to the highest accumulation level of protein in intracellular (IC) or apoplast washing fluid (AWF) fractions of *N. benthamiana* leaf tissues. The addition of 10 units of ‘Ser-Pro’ motifs of hydroxyproline-O-glycosylated peptides (HypGPs) at the C-terminal end of SS^Ext^ mIFNγ (designated SS^Ext^ mIFNγ(SP)_10_) increased the solubility to nearly 2.7- and 1.5-fold higher than those of mIFNγ and SS^Ext^ mIFNγ, respectively. The purified soluble SS^Ext^ mIFNγ(SP)_10_ protein was glycosylated with abundant complex-type N-glycan attached to residues N^56^ and N^128^, and exhibited biological activity against *Sindbis virus* and *Influenza virus* replication in human cell culture systems. In addition, suspension cell cultures were established from transgenic *N. benthamiana*, which produced secreted SS^Ext^ mIFNγ(SP)_10_ protein feasible for downstream processing. These results demonstrate the applicability of the BaMV-based vector systems as a useful alternative for the production of therapeutic proteins, through the incorporation of appropriate fusion tags.

## Introduction

Plants have been developed recently as bioreactors for the industrial-scale production of therapeutic proteins, such as Elelyso^®^ (β-glucocerebrosidase) (Zimran et al., 2015), ZMapp^TM^ (Ebola monoclonal antibodies) (Olinger et al., 2012; Chen and Davis, 2016). Plant-made pharmaceuticals (PMPs) are regarded as safe, with relatively low production cost compared to mammalian systems, and also capable of performing eukaryotic post-translational modifications (PTM) required for biological activity and stability (Pogue et al., 2010; Xu et al., 2012; Bamogo et al., 2019; Schillberg et al., 2019). However, the relatively low yields and the difficulties in downstream processing of PMPs remain major challenges which limit the industrial or commercial acceptance of plant production systems (Schillberg et al., 2019).

To address the low yield issue, we have previously developed a *Bamboo mosaic virus* (BaMV)-based overexpression vector with the co-expression of a silencing suppressor P19, designated pKB19, which was shown to significantly increase the yield of a vaccine candidate in transgenic *Nicotiana benthamiana* suspension cell cultures (Muthamilselvan et al., 2016) or various forms of recombinant human interferon gamma (IFNγ) proteins transiently expressed in inoculated *N. benthamiana* plants (Jiang et al., 2019). However, the downstream processing of the target proteins (TPs) is still challenging. The epitope-presenting chimeric BaMV virions were not designed to be secreted to the medium of the suspension cell cultures (Muthamilselvan et al., 2016), and had to be purified from the cultured biomass. On the other hand, although various strategies applied were effective in increasing the yields of dimeric (D) forms of recombinant interferon gamma IFNγ proteins, such as mature IFNγ (mIFNγ) and mIFNγER (mIFNγ fused with ER retention signal) (Jiang et al., 2019), the lower solubility and lesser N-glycosylation level of the TPs limited their commercial applications as pharmaceuticals. The native human IFNγ is a well-characterized secretory, dimeric glycoprotein (D glycoprotein) generated by the dimerization of two IFNγ monomers with 0, 1, or 2 N-linked glycan each at the two potential glycosylation sites, residues N^25^ and N^97^ (Sareneva et al., 1994, 1995). The N-glycosylation modification is also critical for the half-life of mIFNγ in the bloodstream, affecting its therapeutic usefulness (Hooker and James, 1998; Razaghi et al., 2016). It has been shown that the unglycosylated form of mIFNγ monomers are prone to aggregation through the interactions of the hydrophobic domains, leading to lower solubility (Razaghi et al., 2016) and adding more difficulties for downstream purification processes, such as denaturing and refolding (Jin et al., 2006; Petrov et al., 2010; Razaghi et al., 2017a). Thus, further improvement of the BaMV-based expression system is required for industrial applications.

One of the solutions to the aggregation and glycosylation problems is through the attachment of secretory signals (SSs), guiding TPs along the secretory pathway to extracellular (or apoplast) space, which may simplify the purification process with a higher amount of soluble glycoproteins (Faye et al., 2005; Schneider et al., 2014). Various groups have developed specific SS peptides to improve yields of TP secretion in plants (Becerra-Arteaga et al., 2006; Xu et al., 2007; Benchabane et al., 2009; Huang et al., 2015; Molino et al., 2018). By selecting a specific SS for host species, it has been shown that there could be a 10-fold increase in secretion efficiency of the TPs (Huang et al., 2015; Molino et al., 2018). A glycomodule signal peptide, hydroxyproline (Hyp)-O-glycosylated peptide (HypGP) composed of repeated ‘Ser-Pro’ (SP) or ‘Ala-Pro’ (AP) motif, has been demonstrated to facilitate efficient secretion of many fusion proteins (e.g., IFNα2-(SP)_10,_ human growth hormone GH-(SP)_10_, (SP)_32_-Enhanced green fluorescent protein (EGFP) and human protease inhibitor α1-antitrypsin (AAT)-(AP)_20_) into extracellular space, and dramatically increased yields of TPs in plant cell culture systems (Xu et al., 2007, 2010; Zhang et al., 2016, 2019a). Thus, through fusion of various designer signal peptides to TPs, it is possible to establish a whole-plant or suspension cell culture system to achieve industry-scale and continuous production of valuable therapeutic proteins. However, the efficiencies of the SSs were known to vary widely in different systems, and should be verified for different TPs expressed by various vectors in different plants (Becerra-Arteaga et al., 2006; Huang et al., 2015; Zhang et al., 2016; Molino et al., 2018). It is also worth noting that, although *N. benthamiana* has been extensively used as the model plant system for the production of pharmaceutical proteins, no native signal peptides derived from *N. benthamiana* have been used for the aforementioned purposes in previous studies.

In this study, we aimed to further improve the BaMV-based protein expression system by the incorporation of various plant SSs together with the fusion of secretion booster, (SP)_10_ signal. It was revealed that the fusion of a novel, native SS derived from *N. benthamiana* extensin protein (SS^Ext^) significantly enhanced the solubility, glycosylation, and secretion of the TP, mIFNγ in *N. benthamiana*. In addition, we analyzed the glycosylation modification patterns and verified the biological activity of the fusion proteins. The results revealed that the recombinant IFNγ, namely SS^Ext^ mIFNγ(SP)_10_, expressed by the BaMV-based vector in *N. benthamiana* was glycosylated and exhibited anti-viral activity against *Sindbis virus* (SINV) and *Influenza A virus* (IAV). Furthermore, a suspension cell culture system was established using transgenic *N. benthamiana* for the efficient production of secreted SS^Ext^ mIFNγ(SP)_10_.

## Materials and Methods

### Construction of Recombinant Expression Plasmids

**FIGURE 1:**
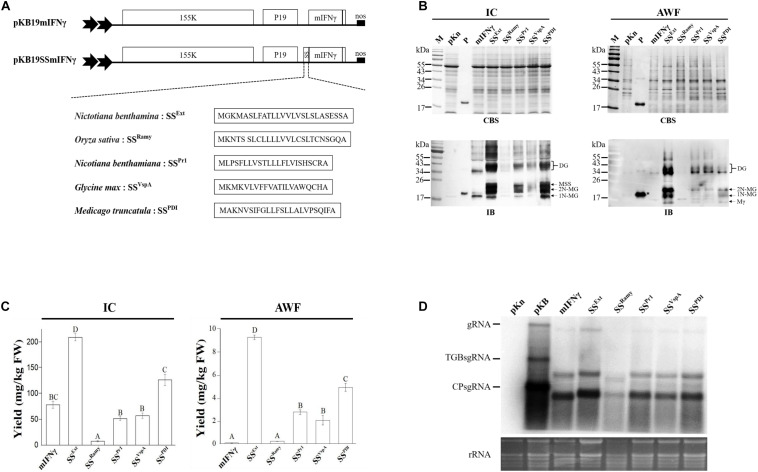
Effects of plant-derived signal peptides on production of mIFNγ glycoproteins in *N. benthamiana* plants. **(A)** Schematic representation of BaMV-based secretory expression cassettes for high-level protein production, secretion and glycosylation. The N-terminus of mIFNγ was fused with one of five different species of plant-derived signal peptides (SS^Ext^, SS^*Ramy*^, SS^*Pr1*^, SS^*Vsp*^ and SS^*PDI*^) as expressed using a BaMV-based vector, pKB19. The open reading frames of this viral vector encode RNA-dependent RNA polymerase (RdRP, 155K) and RNA silencing suppressor P19 from *Tobacco bushy stunt virus* under the control of a dual 35S promoter and a nopaline synthase (*nos*) terminator. **(B)** Analysis of target protein (TP) expression in inoculated leaves at 5 days post-infection (DPI). The *N. benthamiana* leaf exacts were separated into intracellular (IC) and apoplast washing fluid (AWF) fractions, and subsequently analyzed by electrophoresis through a 12% polyacrylamide gel containing 0.1% SDS (SDS–PAGE), followed by staining with Coomassie Blue stain (CBS) and immunoblot (IB) analysis with mIFNγ-specific antibodies. M, marker; pKn, vector only; P, positive control, purified mIFNγ protein derived from *E. coli* (100 ng in CBS and 20 ng in IB) and prepared in TAE buffer; Mγ, M mIFNγ; 1N-MG, M monoglycosylated mIFNγ; 2N-MG, M diglycosylated mIFNγ; MSS, M unprocessed SSmIFNγ; DG, D Glyco-mIFNγ. **(C)** ELISA quantification of mIFNγ levels relative to fresh weight (FW) of leaves (mg/kg) in IC and AWF fractions of infiltrated leaves at 5 DPI. Statistical analysis was performed using one-way analysis of variance (one-way ANOVA) with Tukey’s *post-hoc* multiple comparison analysis. The *P*-value of < 0.001 was considered significantly different, as denoted by different letters. **(D)** Northern blot analysis of wild-type or chimeric BaMV RNA in infiltrated leaves at 5 DPI. BaMV genomic RNA and the subgenomic RNAs for triple gene block proteins (TGPsgRNA) and CP (CPsgRNA) were detected with a BaMV-specific probe, respectively. The bottom panel shows the amount of rRNA in each sample, stained with Ethidium bromide (EtBr) as the loading control.

A previously constructed BaMV expression vector, pKB19mIFNγ (Jiang et al., 2019), containing BaMV RNA-dependent RNA polymerase (RdRp), silencing suppressor P19 (519 bp, GeneBank accession no AJ288926), human mIFNγ (GenBank accession no. AY121833.1) and 6X His-tag under the control of a dual constitutive 35S promoter of CaMV and a nopaline synthase (nos) terminator was used as the starting material for all constructs and also served as the control in the following analyses. It has been reported that fusion of plant-derived SS to N-terminus of TP could TP into secretory pathway and thereby leads to production of secretion protein (Faye et al., 2005; Benchabane et al., 2009). Thus, the strategy was tested on the BaMV-based vector system in this study. Five different plant derived SSs were chosen, including SS^Ext^ from *N. benthamiana* extensin domain (unknown protein IPR006706 in *N. benthamiana* Genome v1.0.1 predicted cDNA), SS^*Ramy*^ from *Oryza sativa* alpha-Amylase (GenBank Accession No. M59351), SS^*Pr1*^ from *N. benthamiana* pathogenesis related protein 1 (GenBank Accession No.: JN247448), SS^*Vsp*^ from *Glycine max* vegetative storage protein (GenBank Accession No. NC038243), and SS^*PDI*^ from *Medicago sativa* protein disulphide isomerase (GenBank Accession No. NC016409) (Benchabane et al., 2009) were constructed by primer extensin of two mutually complementary primer oligonucleotides as listed in Supplementary Table 1, followed by digestion with *Mlu*I. The digestion products were cloned into pKB19mIFNγ to generate the recombinant plasmid pKB19SS^Ext^ mIFNγ, pKB19SS^*Ramy*^mIFNγ, pKB19SS^*Pr1*^mIFNγ, pKB19SS^*Vsp*^mIFNγ, and pKB19SS^*PDI*^mIFNγ, as shown in Figure 1A.

It has been reported that the fusion of a designer HypGP tag composed of ten Ser-Pro dipeptide repeat, (SP)_10_ to the C-terminus of TP leads to dramatically enhanced efficiency of protein secretion in plant cell culture media (Xu et al., 2007, 2010; Ramos-Martinez et al., 2017). Therefore, the strategy was also tested on the viral vector pKB19SS^Ext^ mIFNγ in our study. HypGP tag was constructed by primer extensin of two mutually complementary primer oligonucleotides, F-SP-*Spe*I and R-SP-*Spe*I (Supplementary Table 1), followed by digestion with *Spe*I. The digested product was cloned into pKB19SS^Ext^ mIFNγ to generate the recombinant plasmid pKB19SS^Ext^ mIFNγ(SP)_10_. All constructs were confirmed by sequencing and transformed into *A. tumefaciens* strain pGV3850 via electroporation (Zambryski et al., 1983).

### Transient Expression of TP in *N. benthamiana* Plants

Transient expression in *N. benthamiana* was performed by *Agrobacterium*-mediated infiltration. *A. tumefaciens* (PGV3850) clones harboring different constructs expressing TP were grown separately. *A. tumefaciens* cells were harvested by centrifugation at 12,000 rpm and resuspended in agro-infiltration buffer (10 mM MgCl_2_ and 10 mM MES, pH 5.5) to achieve the appropriate optical density at 600 nm (OD_600_) for each construct. Then the culture was infiltrated into 6-week-old *N. benthamiana* plants (at the 5–6 leaf stage) by the use of a 1 mL a needle-less syringe (OD_600_ = 0.5) or vacuum-infiltration system (10^–1^ to 10^–2^ fold dilutions, of *A. tumefaciens* culture with OD_600_ = 0.5). For vacuum-infiltration, a beaker containing inoculation solution was placed in a vacuum chamber (30 × 20 × 20 cm) with the aerial parts of a plant dipped into the inoculation solution. A vacuum of 25 to 30 mmHg was applied for 1 min. After breaking the vacuum, inoculation solution would be infiltrated immediately into all submerged *N. benthamiana* leaves. The infiltrated plants were maintained in the growth chamber at 28°C, with 16 h light/8 h dark intervals.

### Isolation of TP in IC and AWF Fractions From Infiltrated *N. benthamiana Leaves*

The proteins were isolated from IC and AWF TP fractions as described previously (Alkanaimsh et al., 2016). Agro-Infiltrated fresh leaves were harvested at 5 DPI and submerged in a harvest buffer, TBS-2 (20 mM Tris–HCl, pH 8.0, 150 mM NaCl, 0.02% Silwet L-77) in a glass dish, and subsequently placed in a vacuum chamber for a 1 min vacuum application. The infiltrated leaves were transferred into 50 ml conical-bottom centrifuge tubes, and centrifuged for 15 min at 4°C at 2,500 rpm. The AWF fractions was recovered as the supernatants. One *N. benthamiana* leaf yields 300–400 μl of AWF, which was subsequently 4-fold concentrated through acetone precipitation for silver staining or IB analysis.

### Immunoblotting Assay

Total proteins were extracted from agro-infiltrated leaves at 5 DPI with 1:2.5 (w/v) protein extraction buffer (50 mM Tris–HCl, pH 8.0, 10 mM KCl, 10 mM MgCl_2_, 1 mM EDTA, 20% glycerol, 2% SDS and 10% β-Mercaptoethanol). Extracted total proteins were separated by electrophoresis through a 12% polyacrylamide gel containing 0.1% SDS. The CBS and IB analysis with rabbit primary antibodies against mIFNγ (1:5000 dilution) were used for the determination of TP expression levels of different BaMV-based vectors as described previously (Jiang et al., 2019).

### Northern Blot Analysis

Total RNAs extracted from agro-infiltration leaves at 5 DPI were analyzed using the standard procedure as described (Verwoerd et al., 1989). The accumulation levels of BaMV and chimeric BaMV were hybridized with ^32^P-labeled probes specific to (+)-strand BaMV RNA as described previously (Huang Y.W. et al., 2012).

### Quantitative ELISA

For quantification of TP expression levels of different BaMV-based vectors, ELISA was performed as described previously (Jiang et al., 2019). TSP samples were prepared from inoculated leaves with 1:5 (w/v) ELSIA coating buffer (0.1 M carbonate/bicarbonate buffer, pH 9.6). Following centrifugation at 12,000 × *g* for 10 min, the supernatant was recovered and quantified for TSP using Bradford colorimetric assay (Sigma-Aldrich, St. Louis, MO, United States). The concentration of TSP in each sample was adjusted to approximately 5.5–6.5 mg/mL and 0.8–1.0 mg/mL in IC or AWF, respectively. The 96-well microtiter plates were coated with 5-fold serial dilutions of protein extracts from non-inoculated, inoculated leaves and purified plant-made SS^Ext^ mIFNγ(SP)_10_ or purified mIFNγ protein derived from *E. coli* for standard curve and the positive control. The concentration of TP was determined by comparison with known amounts of the purified mIFNγ protein derived from *E. coli.* All measurements were performed in triplicates.

### Isolation of Soluble SS^Ext^ mIFNγ(SP)_10_ From *N. benthamiana* Extract Homogenates

To obtain soluble TP from *N. benthamiana* leaf homogenates, the vacuum-infiltrated leaves were harvested at 5 to 7 DPI and stored at −80°C as described previously (Hsu et al., 2008). The frozen leaves (20 g) were shredded into small pieces and homogenized with a Polytron^®^ homogenizer at 4°C in 40 ml of extraction buffer A [50 mM Tris–HCl, pH 7.6, 15 mM MgCl_2_ 120 mM KCl, 0.1% β-mercaptoethanol, 20% glycerol, 0.1 mM PMSF, one tablet of cOmplete, EDTA-free proteinase inhibitor cocktails (Roche Life Science, Penzberg, Germany)] (Osman and Buck, 1996). The homogenates were filtered through a layer of Miracloth and centrifuged at 1,000 × g for 10 min at 4°C to separate the pellet (P1) and supernatant (S1) fractions. The P1 contained cell wall, nuclei and chloroplasts of the homogenized cells. The S1 was further centrifuged at 30,000 × *g* for 30 min to separate the membranous (pellet, P30) and soluble (supernatant, S30) fractions. The P30 and S30 samples were subsequently brought to the same volume with protein sample buffer (containing 2% SDS) for CBS and IB analysis.

### Removal of Chloroplasts in S30 Fraction

It has been reported that acetic acid precipitation may enable the removal of the abundant RuBisCO protein for purification of plant-derived protein (Park et al., 2015). The pH of S30 fraction was reduced to 5.1 by treating with ultrapure acetic acid. Then, samples were centrifuged at 30,000 × *g* for 30 min to separate the P30 and S30 fractions.

### The Purification of *N. benthamiana* Expressed SS^Ext^ mIFNγ(SP)_10_

After removal of the abundant chloroplast proteins, the pH of soluble SS^Ext^ mIFNγ(SP)_10_ in S30 fraction was brought to 7.0 by the addition of 1M NaOH, and filtered through a 0.22 μm filter. To obtain pure SS^Ext^ mIFNγ(SP)_10_, the filtrate was loaded on Ni^2+^-NTA column and the SS^Ext^ mIFNγ(SP)_10_ was captured, according to manufacturer’s instructions. The collected eluates were further purified on a HiLoad^TM^ 16/60 Superdex^TM^ 200 pg (S200) column using a fast protein liquid chromatography (FPLC) system (AKTA Purifier GE Healthcare systems) at 4°C in S200 buffer (50 mM Tris–HCl, pH 8.0, 200 mM NaCl, 5 mM β-Mercaptoethanol) following the manufacturer’s instructions. The fractions containing SS^Ext^ mIFNγ(SP)_10_ were collected and concentrated using a Centricon filter (10 kDa NMWL, GE Healthcare, Chicago, IL, United States).

### Glycosylation Analysis of Purified Soluble SS^Ext^ mIFNγ(SP)_10_

The purified plant-made SS^Ext^ mIFNγ(SP)_10_, was glyco-stained using periodic acid-Schiff’s reagent to investigate the glycan moieties, as described previously (Dubray and Bezard, 1982). The N-glycan state of SS^Ext^ mIFNγ(SP)_10_ was examined by deglycosylation analysis using Peptide-N-Glycosidase A (PNGase A) (New England Biolabs, Ipswich, MA, United States) and Peptide-N-Glycosidase F (PNGase F) (New England Biolabs, Ipswich, MA, United States). The plant-made SS^Ext^ mIFNγ(SP)_10_ (0.2 μg), purified from *N. benthamiana*, was denatured and subjected to digestion using 0.5–5 units of PNGase A or PNGase F at 37°C for 48 h, as instructed by the manufacturer. One units is defined as the amount of enzyme required to remove > 95% of the carbohydrate from 1 μg denatured recombinant glycoproteins in 1 h at 37°C.

For determining N-linked glycosylation sites, purified SS^Ext^ mIFNγ(SP)_10_ protein (5 μg) was subjected to in-solution Tryptic/Lys-C digestion based on the filter-aided sample preparation (FASP) protocol reported previously (Wisniewski et al., 2009). The samples were loaded onto 10 kDa filter and treated with 40 μl of ammonium bicarbonate (ABC) with mass spec grade Trypsin/Lys-C (Promega, Madison, WI, United States) at an enzyme : protein ratio of 1:50 (wt/wt) for overnight at 37°C. Trypsin/Lys-C-digested peptide samples were heated at 95°C for 5 min to inactivate enzyme activity, followed by drying in a SpeedVac evaporator. The lyophilized samples were deglycosylated with or without PNGase A (10 unit, purchased from New England Biolabs, Ipswich, MA) for ∼64 hour at 37°C to release the N-glycan. Released glycan were cleaned using C18 cartridges and further analyzed by LC-ESI-MS on a Orbitrap Fusion mass spectrometer (Thermo Fisher Scientific, San Jose, CA, United States) equipped with EASY-nLC 1200 system and EASY-spray source (Thermo Fisher Scientific, San Jose, CA, United States). The digested peptide samples (5 μl) were injected at a flow rate of 1 μl/min onto the column (C18, 0.075 mm X 150 mm, ID 3 μm; Thermo Fisher Scientific, San Jose, CA, United States). LC separation was performed using 0.1% formic acid in water as mobile phase A and 0.1% formic acid in 80% acetonitrile as mobile phase B, operated at a flow rate of 300 nl/min. The gradient employed was 5% buffer B at 2 min to 60% buffer B at 55 min. Full-scan MS condition: mass range m/z 375-1800 (AGC target 5E5) with lock mass, resolution 60,000 at m/z 200, and maximum injection time of 50 ms. The raw data files were processed by MSconvert (v3.0.19246) to generate the peak lists using default parameters. The peak lists were analyzed by Mascot (v2.2.06). The search options used in this study were the user-defined database (SS^Ext^ mIFNγ(SP)_10_ protein sequences only), digestion enzyme trypsin, up to two missed cleavages, fragment ion mass tolerance of 0.6 Da and a parent ion tolerance of 3 ppm. Variable modifications were set to oxidation (M), and carbamidomethyl (C). Peptide ions were filtered using the cut-off score 15. The MS/MS was run in top speed mode with 3 s cycles with CID and HCD; while the dynamic exclusion duration was set to 60 s with a 10 ppm tolerance around the selected precursor and its isotopes. Electrospray voltage was maintained at 1.8 kV and capillary temperature was set at 275°C. For assignment of glycopeptides, the measured m/z values were used to search against a database that combines predicted tryptic peptides (by Protein Digest Simulator Basic) and N-linked glycan (from Consortium for Functional Glycomics) by in-house software (Liu et al., 2011). The assigned glycopeptides were manually confirmed by the appearance of glycan fragments in MS/MS spectra (Liu et al., 2011).

### Recombinant *Sindbis Virus* Infection and IFNγ Treatment

Recombinant *Sindbis virus* (SINV-eGFP) containing an enhanced green fluorescent protein (eGFP) expression cassette was kindly provided by Lih-Hwa Hwang (Graduate Institute of Microbiology and Immunology, National Yang Ming University, Taipei, Taiwan) (Huang P.Y. et al., 2012). In order to test the effect of human IFNγ (hIFNγ) against recombinant SINV-eGFP infection, the HEK 293T cells (2.5 × 10^5^) were seeded in 24-well plate. At 8 h after seeding, the cells were washed twice with PBS and then treated with either DMEM (Mock), the extract from *N. benthamiana* healthy leaves as the negative control (N), or a series of concentrations of PC-IFNγ (R&D Systems, Inc., MPLS, United States) as well as SS^Ext^ mIFNγ(SP)_10_ for 12 h at 37 °C with 5% CO_2_. Subsequently, cell monolayer was infected with SINV-eGFP virus at a multiplicity of infection (M.O.I) of 1.0. The infected cells were collected at 24 h post-infection (hpi). The levels of eGFP signals and viral proteins were determined by fluorescence microscope observation and IB analysis with rabbit primary antibodies against eGFP, respectively.

### *Influenza Virus* Infection and Interferon Gamma Treatment

*Influenza virus* subtype H1N1, strain A/Puerto Rico/8/34 (PR8) was kindly provided by Paul Digard (University of Edinburgh, United Kingdom). In order to test the activity of hIFNγ against PR8 (H1N1), MDCK cells (4 × 10^4^/well) were seeded in 24-well plate for 8 h prior to treatment. Cells were washed twice with PBS and treated with DMEM, the extracts of *N. benthamiana* healthy leaves (N), or various concentrations of PC-IFNγ and SS^Ext^ mIFNγ(SP)_10_. After 12 h incubation, cell monolayer was infected with PR8 at an M.O.I of 1.0 for 24 hpi. The accumulation level of PR8 viral proteins NS1 and NP were determined by IB analysis with mouse primary antibodies against actin (1:2500 dilution), or chicken primary antibodies against NS1 or NP (1:2500 dilution).

### *N. benthamiana* Suspension Cells Culture

**FIGURE 2:**
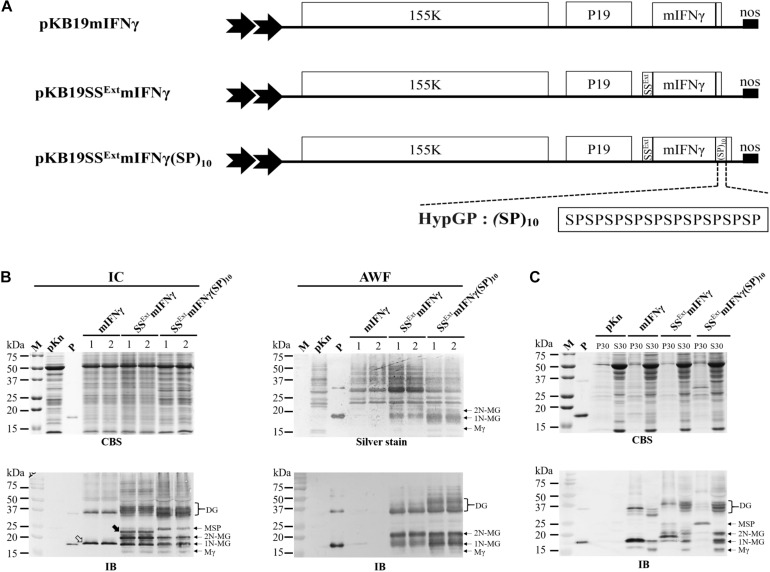
Effect of (SP)_10_ signal on glycoprotein accumulation and solubility. **(A)** Schematic representation of BaMV-based secretory expression cassettes with the C-terminal end of SS^Ext^ mIFNγ fused with 10 units of ‘Ser-Pro’ motifs of hydroxyproline (Hyp)-O-glycosylated peptides (HypGPs) (designated SS^Ext^ mIFNγ(SP)_10_). **(B)** Analysis of TP expression in IC and AWF fractions at 5 DPI by SDS–PAGE, followed by staining with CBS (for IC fraction) or silver stain (for AWF) and IB analysis with alkaline phosphatase (AP)-conjugated mIFNγ-specific antibodies. Lanes 1 and 2, refer to two independent experiments, each performed in triplicate. The unprocessed mIFNγ and SS^Ext^ mIFNγ products are indicated by the blank and solid arrowhead, respectively. **(C)** Analysis of the solubility of TP. The protein extracts of *N. benthamina* leaf (20 g) without SDS detergent in extraction buffer A were separated into membranous fraction (P30) and soluble fraction (S30) after centrifugation at 30,000 × *g*, and subsequently analyzed by SDS–PAGE, followed by staining with CBS and IB analysis. The positions of various forms of SS^Ext^ mIFNγ(SP)_10_ proteins are indicated on the right, based on the estimated molecular weights. M, marker; pKn, vector only; P, positive control, purified mIFNγ protein derived from *E. coli*; Mγ, M mIFNγ; 1N-MG, M monoglycosylated mIFNγ; 2N-MG, M diglycosylated mIFNγ MSP, M unprocessed SS^Ext^ mIFNγ(SP)_10_; DG, D Glyco-mIFNγ.

Leaf disks of *N. benthamiana* were transformed with *A. tumefaciens* harboring pKB19mIFNγ and pKB19SS^Ext^ mIFNγ(SP)_10_ constructs (Figure 2A) to generate transgenic *N. benthamiana* plants as described previously (Horsch et al., 1985; Muthamilselvan et al., 2016). The putative transgenic *N. benthamiana* (R0) were screened by PCR and IB analysis. Homozygous individuals were selected from F1 to F2 progenies on the basis of kanamycin resistance. All homozygous F2 progenies of transgenic *N. benthamiana* lines were further selected for TPs expression by IB analysis.

Four F2 transgenic lines expressing SS^Ext^ mIFNγ(SP)_10_, 3-6-5, 3-9-4, 4-5-1, and 4-14-9 were used for the establishment of suspension cell cultures. The wild-type (non-transgenic) and F2 transgenic *N. benthamiana* expressing mIFNγ, line 50-1-3, were used as the negative and positive control, respectively. Leaf explants of the aforementioned plants were cultured and induced on MS callus selection medium plates (Duchefa Biochemie, Haarlem, Netherlands) as described previously (Muthamilselvan et al., 2016). The subcultures were carried out at a 14-day interval and whitish callus tissues formed on leaf explants in 7–8 weeks. In order to generate transgenic suspension cells, approximately 1 g friable callus biomass was transferred to 25 mL Schenk Hildebrandt (SH) liquid media (1:5 of volume ratio), and rotated at 120 rpm on a gyratory shakers at 25–26°C. For kinetic studies of the cell growth and protein secretion, suspension cultured cells were collected at an interval of 2 days for determination of cell biomass and secreted SS^Ext^ mIFNγ(SP)_10_.

### Statistical Analysis

Statistical analysis was performed using the one-way analysis of variance (one-way ANOVA) with Tukey’s *post hoc* multiple comparison analysis (SPSS version 19, IBM Corp, Armonk, NY, United States). Mean values and SD from three independent experiments with technical triplicates are presented. *P*-values < 0.001 were considered significant.

## Results

### Modification of BaMV-Based Secretory Cassettes by Incorporating Various Signal Sequences

To enhance the solubility, glycosylation, and secretion of the mIFNγ produced through the previously constructed BaMV-based vector, pKB19, five different plant-derived SS candidates, namely SS^Ext^ (this study), SS^*Ramy*^ (Alkanaimsh et al., 2016), SS^*Pr1*^ (Zhu et al., 2012), SS^*Vsp*^ (Becerra-Arteaga et al., 2006), and SS^*PDI*^ (Benchabane et al., 2009) were individually fused to the N-terminus of mIFNγ (Figure 1A). It should be noted that the SS^Ext^ used in this study was derived from *N. benthamiana* extensin protein, which is distinct from those of *N. tabacum* (Xu et al., 2007; Xu et al., 2010) and *N. plumbaginifolia* (De Loose et al., 1991) (Supplementary Figure 1) shown to facilitate the secretion of TPs in *N. tabacum* BY-2 cells. In addition, although SS^*Pr1*^ is derived from *N. benthamiana*, it has not been assayed for the function in enhancing the solubility or secretion efficiency of TPs. These expression cassettes in pKB19 were constructed under the control of dual constitutive 35S promoter of *Cauliflower mosaic virus* (CaMV) and a nopaline synthase (nos) terminator (Figure 1A). To compare the efficiency of various signal sequences, *A. tumefaciens* cells harboring individual construct were infiltrated into *N. benthamiana* leaves. The transiently expressed TPs were harvested and analyzed at the fifth day post inoculation (DPI) as described below.

### Fusion of Plant-derived SS to N-terminus of mIFNγ Led to the Increased Secretion of Glycosylated mIFNγ

To verify whether different SSs could guide TP through the secretory pathway, the extracts from infiltrated leaf tissues were separately processed as intracellular (IC) or apoplast washing fluid (AWF) fractions by vacuum as described in the section “MATERIALS AND METHODS”, and subsequently assayed for protein yields using immunoblotting (IB) analysis and ELISA with mIFNγ-specific antibodies. The result of IB analysis of five SSmIFNγ protein samples in AWF fraction (Figure 1B, right panel) revealed the presence of monomeric mIFNγ (Mγ), monomeric monoglycosylated mIFNγ (M Glyco-mIFNγ, 1N-MG) and monomeric diglycosylated mIFNγ (2N-MG) with relative molecular masses (*M*_*r*_) of 16, 18, and 20 kDa, respectively, resulting from the proteolytic cleavage of both SSs and C-terminal polypeptide of SSmIFNγ with a difference in the degree of N-glycosylation as observed previously (Curling et al., 1990; Sareneva et al., 1994; James et al., 1996). The dimeric Glyco-mIFNγ (DG) with apparent *M*_*r*_ of 36–40 kDa was also observed, presumably resulted from the dimerization of Glyco-mIFNγ. (Figure 1B, right panel) (Sareneva et al., 1994). In contrast, little or no TPs were detected in the AWF fraction of the control mIFNγ sample by IB analysis (Figure 1B, right panel), indicating that the fusion of various SS peptides could lead to the production of secreted mIFNγ. The protein extracts isolated from IC fraction were also analyzed by IB with mIFNγ-specific antibodies. The result showed the existence of TPs similar to those observed in the AWF fraction, including post-translationally processed 1N-MG, 2N-MG and DG with apparent *M*_*r*_ of 18, 20, and 36–40 kDa, respectively (Figure 1B, left panel). An additional protein with apparent *M*_*r*_ of 23 kDa was also observed (Figure 1B, lower left panel, MSS), which may represent the unprocessed M SSmIFNγ, retaining SS polypeptide. In contrast, only the M and D unprocessed forms of mIFNγ with apparent *M*_*r*_ of 18 and 36 kDa, respectively, were detected in the mIFNγ control sample. This result demonstrated that the fusion of plant-derived SSs may facilitate the production and secretion of processed SSmIFNγ glycoproteins through the secretory pathway toward apoplast in plants.

The overall accumulation of mIFNγ fused with various SSs and the control mIFNγ in IC or AWF fractions were further quantified using ELISA (Figure 1C). The result showed that the SS^Ext^ fusion led to the highest level of TPs accumulation among the five SSmIFNγ constructs, up to 209 ± 7 mg/kg fresh weight in IC and 9.5 ± 0.45 mg/kg in AWF, reaching an almost 2.7-fold and 92.5-fold compared to that of the control mIFNγ in IC and AWF, respectively. However, the accumulation level of SS^*Ramy*^ mIFNγ was the lowest (Figure 1C, right panel), with almost no detectable TP in AWF fraction by IB analysis (Figure 1B, right panel). For comparison of BaMV RNA accumulation levels of different constructs, total RNAs were extracted and analyzed by northern blot using BaMV-specific probes. The result revealed that BaMV RNA also accumulated to the highest level in leaves infiltrated with SS^Ext^ mIFNγ construct at 5 DPI as compared to those infiltrated with other BaMV expression cassettes, suggesting that the fusion of different SS coding sequences may affect the accumulation levels of the viral vectors (Figure 1D). The above results indicated that the native SS^Ext^ derived from *N. benthamiana* facilitated significant enhancement of secretion and yields of TP in the current expression system.

### Additional C-terminal Fusion of (SP)_10_ Further Enhanced TP Yield and Solubility

In order to improve the secretion and solubility of TP, the C-terminal end of SS^Ext^ mIFNγ was fused with 10 units of ‘Ser-Pro’ motifs of HypGP tag (designated SS^Ext^ mIFNγ(SP)_10_) (Figure 2A). IB analysis of IC- or AWF-fraction protein extracts from *N. benthamiana* leaves infiltrated with *A. tumefaciens* harboring pKB19SS^Ext^ mIFNγ(SP)_10_ construct revealed the presence of specific bands at 16 kDa (Mγ), 18 kDa (1N-MG), and 20 kDa (2N-MG) in both IC and AWF fractions (Figure 2B), which might be generated from post-translational modification by the proteolytic cleavage of SS^Ext^ and the C-terminal (SP)_10_ peptides from SS^Ext^ mIFNγ(SP)_10_, with a difference in the degree of N-glycosylation. The dimeric Glyco-mIFNγ (DG) with apparent *M*_*r*_ of 36–40 kDa was also observed, presumably resulted from the dimerization of Glyco-mIFNγ with or without the C-terminal His-tag of the partially O-glycosylated forms, as estimated by their relative molecular weights. However, the identities of these proteins await further analysis. (Figure 2B). An extra protein was observed with an apparent *M*_*r*_ of 25 kDa, which may be unprocessed M SS^Ext^ mIFNγ(SP)_10_, retaining SS and (SP)_10_ polypeptides in IC fraction (Figure 2B, lower left panel, MSP). Three distinct protein bands were detected by silver staining only in the AWF fractions from leaves infiltrated with *A. tmafaciens* harboring the pKB19SS^Ext^ mIFNγ and pKB19SS^Ext^ mIFNγ(SP)_10_ constructs, with apparent *M*_*r*_ of 16 kDa (Mγ), 18 kDa (1N-MG) and 20 kDa (2N-MG) (indicated on the right of the panel, Figure 2B, upper right), which provided further evidence in support that the TPs were secreted out of plant cells. The overall accumulation levels of TPs in IC and AWF were further quantified using ELISA. The result revealed that the average levels of TPs produced in leaves infiltrated with *A. tmafaciens* harboring pKB19SS^Ext^ mIFNγ(SP)_10_ construct were significantly increased, up to 489 ± 28 mg/kg and 15.2 ± 0.98 mg/kg fresh weight in IC or AWF, respectively (Table 1). The result indicated that the fusion of (SP)_10_ tag could further enhance the yield of TPs.

**TABLE 1:** Expression levels of mIFNγ, SS^Ext^ mIFNγ and SS^Ext^ mIFNγ(SP)_10_ in IC or AWF as quantified by ELISA.

**Constructs**	**IC^†^**		**AWF^‡^**	
	**Yield (mg/kg FW)^§^**	**%TSP^¶^**	**Yield (mg/kg FW)^§^**	**%TSP^¶^**
mIFNr	78^*A*^ ± 7	1.4	0.3^*a*^ ± 0.06	0.03
SS^Ext^ mIFNγ	209^*B*^ ± 7	3.5	9.5^*b*^ ± 0.45	1.1
SS^Ext^ mIFNγ(SP)_10_	489^*C*^ ± 28	7.5	15.2^*c*^ ± 0.98	1.7

*Statistical analyses were performed using one-way analysis of variance (one-way ANOVA) with Tukey’s *post hoc* multiple comparison analysis. Mean values with dissimilar superscripts (A,B,C for IC and a, b, c for AWF) are significantly different at *P*-value < 0.001 level. Abbreviations: ^†^ IC, Intercellular space; ^‡^ AWF, Apoplast washing Fluid; ^§^ FW, fresh weight; ^¶^ TSP, total soluble protein.*

To examine whether the fusion of SS^Ext^ and (SP)_10_ to mIFNγ could increase the solubility of the recombinant protein SS^Ext^ mIFNγ(SP)_10_, total protein extracts from *Agrobacterium* vacuum-infiltrated leaves were separated into the precipitated membranous (P30) and soluble (S30) factions following a two-step centrifugation process at 1,000 and 30,000 × *g*. Subsequently, the protein samples were analyzed by IB with mIFNγ-specific antibodies. It was found that the accumulation levels of Mγ (16 kDa), 1N-MG (18 kDa), 2N-MG (20 kDa) and DG (36–40 kDa) were higher in the soluble fraction (S30) from leaves expressing SS^Ext^ mIFNγ(SP)_10_ as compared to those from leaves expressing mIFNγ or SS^Ext^ mIFNγ (Figure 2C). In contrast, in the P30 fraction, two specific bands were observed with apparent *M*_*r*_ of 25 and 50 kDa, likely to be M and D unprocessed SS^Ext^ mIFNγ(SP)_10_, respectively (Figure 2C), representing precursors targeted to endoplasmic reticulum (ER) before being translocated into the secretory pathway. To estimate the amounts of soluble proteins in different samples, each specific band in S30 and P30 fractions was further qualified by densitometry. The result revealed that the percentage of soluble TPs produced in leaves expressing SS^Ext^ mIFNγ(SP)_10_ was increased up to 81%, which is much higher than those from leaves infiltrated with the mIFNγ (30%) or SS^Ext^ mIFNγ (56%), corresponding to a 2.7- or 1.5-fold increase, respectively (Figure 2C, IB). In comparison, the TPs produced by the construct pK19mIFNγER in our previous study were targeted into the ER compartment and partitioned mostly to P30 fraction even though various detergents were used in an attempt to solubilize the TPs in the purification processes (Supplementary Figures 2A,B). These results indicated that, by fusion with the plant derived signal peptides SS^Ext^ and (SP)_10_ tag, the yields and solubility of the TPs, such as mIFNγ, expressed by using BaMV-based vectors could be greatly enhanced.

### Purification of SS^Ext^ mIFNγ(SP)_10_ Protein Through Acid Precipitation Coupled With Ni^2+^-NTA and Gel Filtration Chromatography

Reduction of non-TPs is an important issue for the production process. As seen in Figure 2C, the Ribulose-1,5-bisphosphate carboxylase/oxygenase (RuBisCO) large subunit could be observed (53 kDa) as the predominant contaminating protein in the S30 fraction extracted from vacuum-infiltrated leaves. There have been several methods developed for the removal of RuBisCO proteins, with various degrees of success and concerns (Boyhan and Daniell, 2011; Gupta et al., 2015; Park et al., 2015). To reduce the contaminating RuBisCo protein level and obtain SS^Ext^ mIFNγ(SP)_10_ proteins with a higher purity, the S30 fraction was processed by the acetic acid method (Park et al., 2015) and further purified through Ni^2+^-NTA and gel filtration chromatography. The resultant fractions were analyzed by SDS-PAGE and the TPs were visualized by CBS and IB (Supplementary Figure 3). Various forms of mIFNγ were detected in the eluates following the addition of 500 mM imidazole, including Mγ (16 kDa), 1N-MG (18 kDa) and 2N-MG (20 kDa) and DG (36–40 kDa) (Supplementary Figure 3, lane Ni^2+^-NTA). In addition, the RuBisCO contaminations in the S30 fraction could be removed through acetic acid precipitation coupled with Ni^2+^-NTA chromatography (Supplementary Figure 3, lane Ni^2+^-NTA). The imidazole-eluted fractions were collected and subject to gel filtration column (Superdex^TM^ 200 pg, S200) for further purification. The gel filtration chromatography profiles revealed the presence of different forms of mIFNγ proteins in two major peaks, corresponding to fractions 13-23 and 25-63, respectively (Supplementary Figures 4A,B). Fractions 25–63 were further concentrated by centrifugal ultrafiltration disks (10 kDa NMWL, GE Healthcare, Chicago, IL, United States) to increase protein purity (Supplementary Figure 3, lane S200). The purified SS^Ext^ mIFNγ(SP)_10_ and TSP were subjected to quantitative ELISA and Bradford assay. The results showed that the purity of TP was increased up to 91% and the final yield was estimated to be approximately 94 ± 7 mg/kg tissue weight (FW) as summarized in Table 2, which is comparable to PMPs production in certain plant-based bioreactors (Chan and Daniell, 2015; Mortimer et al., 2015).

**TABLE 2:** The purification of SS^Ext^ mIFNγ(SP)_10_ by acetic acid precipitation coupled with Ni^2+^-NTA and gel filtration chromatography^†^.

	**N^‡^**	**SS^Ext^ mIFNγ(SP)_10_**		
**Fractions**	**S30**	**S30**	**Ni^2+^NTA**	**S200^§^**
Yield (mg/kg)	0	551 ± 43	326 ± 41	94 ± 7
TSP (mg/kg) ^¶^	3825 ± 202	3735 ± 34	455 ± 45	103 ± 8
Purity (%)	–	15	72	91

***^†^**: The yields of different fractions through the purification process were determined by ELISA with specific antiserum against mIFNγ. Abbreviations: ^‡^ N, healthy leaf extract as a negative control; ^§^ S200, gel filtration chromatography (Superdex^TM^ 200 pg); ^¶^ TSP, total soluble protein.*

### Plant Made SS^Ext^ mIFNγ(SP)_10_ Is Glycosylated

**FIGURE 3:**
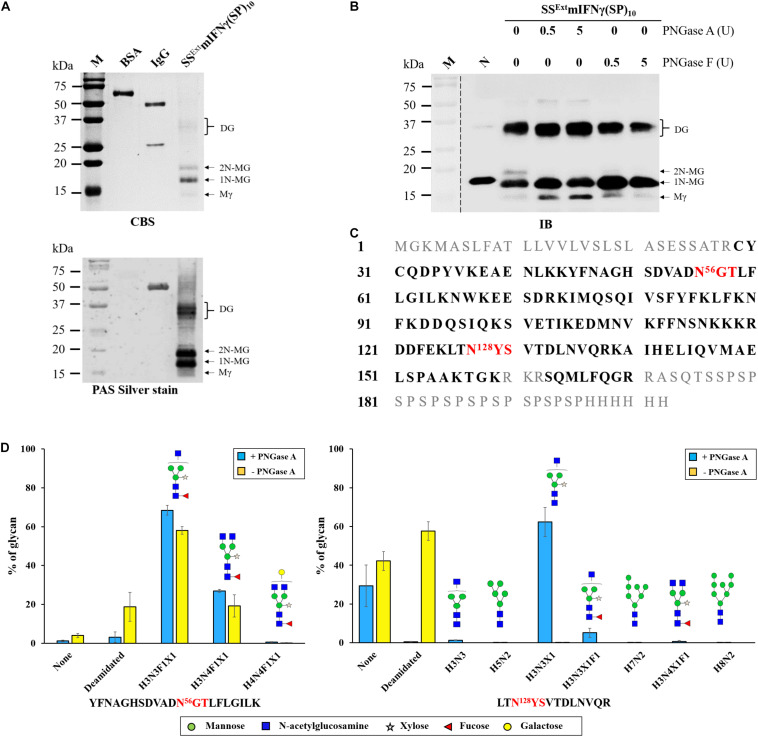
Analysis of the glycosylation of SS^Ext^ mIFNγ(SP)_10_. **(A)** Glycoprotein analysis of SS^Ext^ mIFNγ(SP)_10_ by CBS and Periodic acid-Schiff (PAS) silver stain. Protein samples (0.2 μg each) were separated by SDS–PAGE as described above and examined by CBS or PAS. The identity of each sample is indicated on top of each lane. BSA and IgG from 33D2 monoclonal antibody were used as the negative and positive control, respectively. **(B)** Deglycosylation analysis of SS^Ext^ mIFNγ(SP)_10_ with peptide-N-glycosidase A and F (PNGase A and F) treatment. The elimination of N-glycan of purified SS^Ext^ mIFNγ(SP)_10_ was detected by IB analysis after PNGases (0.5–5 units) digestion. mIFNγ derived from *E. coli* as a negative control. SS^Ext^ mIFNγ(SP)_10_ was purified from *N. benthamiana*. The “–” and “+” signs represent treatments with or without PNGases. Various forms of SS^Ext^ mIFNγ(SP)_10_ proteins were indicated on the right. **(C)** The amino acid sequence of SS^Ext^ mIFNγ(SP)_10_ mapped by LC-MS/MS-based peptide sequencing. Following in-solution tryptic digestion, the samples were subjected to LC-MS/MS analysis. The resulting amino acid sequence covering 68% of mIFNγ (29–170 residues) were identified and shown in bold. No peptides were identified for the SS^Ext^ (residues 1–26) and (SP)_10_ and 6X His (residues 171-202) regions of the amino sequence, shown in gray. Predicted N^56^ and N^128^ glycosylation sites were shown in red. **(D)** Tryptic digested glycopeptides of residues 45–65, YFNAGHSDVADN^56^GTLFLGILK, and residues 126–138, LTN^128^YSVTDLNVQR, from the SS^Ext^ mIFNγ(SP)_10_ were identified for glycosylation composition by LC-MS/MS analysis. The percentage of each glycan among the total signals is shown. The white, green, orange and blue columns represent the percentage of non-glycosylation, mannose-, hybrid- or complex-type glycan, respectively. The abbreviations used for monosaccharides are: H, hexose; N, N-acetylhexosamine; F, fucose; X, xylose.

The authentic hIFNγ is a secretory glycoprotein which has two potential N-linked glycosylation sites (Sareneva et al., 1994, 1995) and three glycoprotein forms: non-glycosylated (Mγ); monoglycosylated at N^25^ (1N-MG), and diglycosylated at N^25^ and N^97^ (2N-MG) (Hooker and James, 1998). In order to investigate the glycosylation state of plant-derived SS^Ext^ mIFNγ(SP)_10_, both periodic acid-Schiff stains (PAS) (Dubray and Bezard, 1982) and enzymatic deglycosylation of PNGase A (Takahashi, 1977; Wang et al., 2018) or PNGase F (Tarentino et al., 1985) methods were performed. The purified TP described above was subjected to SDS-PAGE followed by PAS silver staining (Figure 3A). The result revealed the presence of different forms of mIFNγ with specific glycosylation patterns in contrast to that of the BSA protein as the negative control. The different forms of Glyco-mIFNγ were further digested with PNGase A or PNGase F, followed by IB with mIFNγ-specific antibodies to verify whether these were N-glycosylated as the authentic hIFNγ does. The PNGase F-based deglycosylation enables cleavage of the asparagine-linked complex, hybrid, or high mannose oligosaccharides. However, the α-(1,3)-fucose attached on the core glycan N-linked to glycoproteins produced in plant can only be removed specifically with PNGase A (Bardor et al., 2006). The IB result showed that the 2N-MG (20 kDa) and DG (36–40 kDa) were digested by PNGase F treatment, migrating faster into specific positions at 18 kDa and 35–36 kDa, respectively (Figure 3B). In contrast, deglycosyaltion of Glyco-mIFNγ with PNGase A resulted in the alteration of the Mγ : 1N-MG ratio, with the increase of the Mγ (16 kDa) relative to that of 1N-MG (18 kDa), as compared to those treated with PNGase F or no enzyme (Figure 3B). The result suggested that Glyco-mIFNγ was only partially deglycosyated by PNGase A, which was in accordance with the previous reports (Mishra et al., 2006; Wang et al., 2018). The observation indicated that 1N-MG, 2N-MG or DG were modified by N-glycosylation.

It has been shown that recombinant hIFNγ proteins produced in other plant platforms were glycosylated as well (Chen et al., 2004; Castilho et al., 2018; Heidari-Japelaghi et al., 2020), but the glycosylation profiles have not been identified in these previous studies. To characterize the putative glycosylation sites and glycan profiles, the Tryptic/Lys-C digested plant-made SS^Ext^ mIFNγ(SP)_10_ was treated with or without PNGase A and further analyzed by liquid chromatography-tandem mass spectrometry (LC-MS/MS). Two N-glycosylated peptides were detected and identified from their molecular masses as being residues (45–65) YFNAGHSDVADN^56^GTLFLGILK and residues (126–138) LTN^128^YSVTDLNVQR, containing putative N-glycosylation sites at N^56^ and N^128^ (Figures 3C,D), which corresponded to N^25^ (with fucosylated complex-type oligosaccharides) and N^97^ (with non-fucosylated hybrid- and high-mannose-type structures) of the native mature hIFNγ glycosylation sites, respectively (Sareneva et al., 1996; Razaghi et al., 2016). The assignment of the respective N-glycan structures was shown in Supplementary Figures 5–14. The most abundant glycan detected at the N^56^ site was of the α-(1,3)-fucosylated complex-type structure, ∼68% MGnXF (H3N3F1X1) and ∼27% GnGnXF (H3N4F1X1), whereas the major glycan attached to the N^128^ site was the non-fucosylated complex-type MGnX (H3N3X1, ∼62%). Both glycosylation sites at N^56^and N^128^, the major proportion of N-glycan were complex-type structures, indicating that, during the transportation through the secretory pathway, the TP was fully glycosylated as expected. After PNGase A treatment, most of heterogeneous glycan at the N^128^ glycosylation site could be completely released. In contrast, only 19.3% α-(1,3)-fucose of the complex-type glycan (accounting for 96% of total glycan) at N^56^ site was released by PNGase A. This observation was in accordance to the result of IB analysis of deglycosylated SS^Ext^ mIFNγ(SP)_10_, showing only partial digestion after PNGase A treatment. In addition, approximately 68% coverage of the major mIFNγ sequence (residues 29–170, approximately 16 kDa, as predicated based on *M*_*r*_) was mapped from the purified plant-made SS^Ext^ mIFNγ(SP)_10_ sample, whereas the SS (residues 1–26), C-terminal end (residues 171–202, containing mIFNγ residues of Arg^171^- Ala^172^-Ser^173^-Gln^174^, (SP)_10_ and 6X His-tag) peptides were not detected by the LC-MS/MS-based sequencing (Figure 3C). The result also suggested that the majority of plant-made SS^Ext^ mIFNγ(SP)_10_ were truncated to approximately the same extent as *Spodoptera frugiperda* (Sf9)-derived hIFNγ (terminating at Gly-Arg-Arg region) (James et al., 1996). The above observation indicated that Mγ (16 kDa) was possibly the processed products lacking plant-derived SS and (SP)_10_ signals through proteolytic cleavage at C-terminus of SS^Ext^ mIFNγ(SP)_10_ molecules and further N-glycosylated for the production of both 1N-MG (18 kDa) and 2N-MG (20 kDa) forms during the transportation from ER through the trans-Golgi network.

### SS^Ext^ mIFNγ(SP)_10_ Is Biologically Active in Suppressing Viral Replication

To test the biological activity of purified SS^Ext^ mIFNγ(SP)_10_, the antiviral effects was analyzed using a recombinant *Sindbis virus* expressing eGFP (SINV-eGFP) as the target pathogen on cultured HEK 293T cells (Huang P.Y. et al., 2012). Culture medium (DMEM) was first supplemented with various concentrations of SS^Ext^ mIFNγ(SP)_10_ ranging from 0.5 ng to 50 ng 12 h prior to SINV-eGFP infection at M.O.I of 0.1. In this assay, commercial interferon (PC-IFNγ), and protein extract from healthy *Nicotiana benthamiana* (N) at the same range of concentrations were used as positive and negative control, respectively. At 24h post infection (hpi), the GFP fluorescence, representing overall infection status, was initially observed by fluorescent microscopy. As shown in Supplementary Figures 15A,B, upon treatment with 5 or 50 ng SS^Ext^ mIFNγ(SP)_10,_ the GFP fluorescence signals were confined to single cell and apparently decreased, compared with that in negative controls N (50 ng) and BSA (500 ng).

**FIGURE 4:**
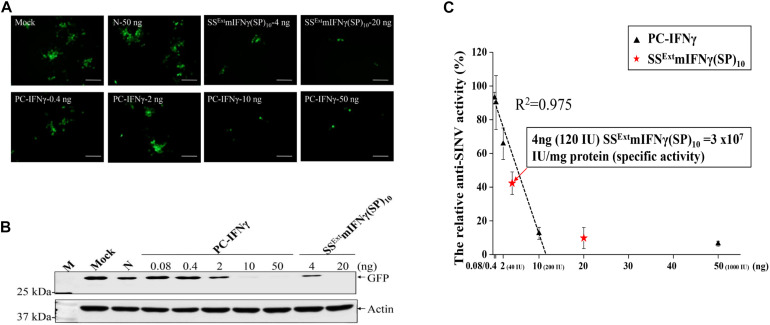
Biological activity of SS^Ext^ mIFNγ(SP)_10_ against SINV. HEK293-T cells were either pre-incubated with DMEM (Mock), 50 ng of protein extracts from *N. benthamiana* healthy leaves as negative control (N), or different concentrations of positive control (PC, commercial IFNγ) or SS^Ext^ mIFNγ(SP)_10_, as indicated in the panel, for 12 h. Subsequently, cells were infected with SINV-eGFP at an M.O.I of 1.0, and analyzed at 24 hpi. **(A)** eGFP signals as observed by fluorescence microscopy. Scale bar = 100 μm. **(B)** The accumulation level of eGFP and actin as analyzed by IB analysis. **(C)** The relative anti-viral activity of SS^Ext^ mIFNγ(SP)_10_. The standard curve of anti-viral effect was initially established by quantifying the intensity of eGFP signals in cells pre-treated with various concentrations of PC-IFNγ (with specific activity of 2X10^7^ IU/mg protein). The relative activity of SS^Ext^ mIFNγ(SP)_10_ was then estimated using the standard curve.

Next, the relative activity of SS^Ext^ mIFNγ(SP)_10_ was evaluated using PC-IFNγ as the standard. The PC-IFNγ with specific activity of 2 X 10^7^ IU/mg was 5-fold serially diluted from 50 ng to 0.4 ng. The eGFP fluorescence intensity was apparently reduced upon treatment of either PC-IFNγ or SS^Ext^ mIFNγ(SP)_10_, as compared with N (Figure 4A). The overall eGFP accumulation level, as measured by IB analysis, was also dramatically decreased by PC-IFNγ treatment in a dose-dependent manner (Figure 4B). On the basis of eGFP accumulation level (Figure 4B), a standard curve of anti-viral activity mediated by PC-IFNγ was established (Figure 4C). Based on the standard curve, the specific activity of 4 ng SS^Ext^ mIFNγ(SP)_10_ was determined to be approximately 120 IU ( = 3 × 10^7^ IU/mg protein) (Figure 4C), which was comparable to that of the PC-IFNγ ( = 2 × 10^7^ IU/mg protein). These results demonstrated that plant-made SS^Ext^ mIFNγ(SP)_10_ protein exhibits anti-viral biological activity and is able to prevent the spread of SINV-eGFP to neighboring cells.

**FIGURE 5:**
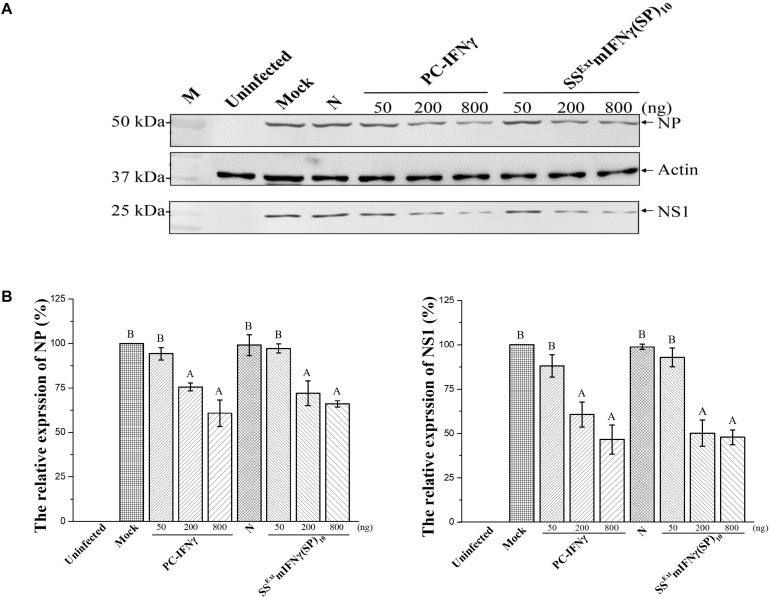
Biological activity of SS^Ext^ mIFNγ(SP)_10_ in suppressing IAV replication. MDCK cells were left uninfected or pre-incubated with DMEM (Mock), 800 ng of protein extracts from *N. benthamiana* healthy leaves as the negative control (N), or three different concentrations (50, 200, 800 ng) of PC- IFNγ, or SS^Ext^ mIFNγ(SP)_10_, for 12 h followed by infection with IAV at an M.O.I of 1.0. **(A)** Western blot analysis of viral protein expression levels. At 24 hpi, total cells were harvested and subjected to western blot analysis for the determination of expression of viral proteins NS1 and NP. The amount of actin was used as the internal loading control. **(B)** Quantification of protein levels of NS1 and NP by densitometry. The accumulation level of viral proteins in DMEM (Mock) treated cells was arbitrarily set as 100% and the relative protein accumulation levels in N, PC-IFNγ, and SS^Ext^ mIFNγ(SP)_10_ relative to Mock was plotted. Statistical analysis was performed using one-way ANOVA with Tukey’s *post hoc* multiple comparison analysis. Mean expression ratio (%) and SD from three independent experiments with technical triplicates are presented. The *P*-value of < 0.001 was considered significantly different, as denoted by different letters.

Another biological activity of mIFNγ is to suppress the replication of certain viruses, including *Influenza virus* (IAV). The induction of endogenous mIFNγ secretion inhibited IAV (subtype H1N1) infection in MDCK cells (Hu et al., 2016). Thus, we further evaluated the effect of purified recombinant SS^Ext^ mIFNγ(SP)_10_ protein on the replication of IAV (H1N1, PR8 strain). MDCK cells were cultured in DMEM (Mock) with various concentrations of SS^Ext^ mIFNγ(SP)_10_ ranging from 50–800 ng for 12 h followed by challenging with IAV H1N1. The accumulation levels of viral proteins NP and NS1 were monitored at 24 hpi. Similar to that of PC-IFNγ, the overall accumulation of both viral proteins were reduced in cells pre-treated with high concentrations of SS^Ext^ mIFNγ(SP)_10_ (Figure 5A). Quantification of the accumulation levels of NP and NS1 further revealed that the high-dose (i.e., 200 and 800 ng) treatment of SS^Ext^ mIFNγ(SP)_10_ significantly decreased the amount of NP (*P*-values < 0.001) and NS1 (*P*-values < 0.001), as shown in Figure 5B. Our results indicate that pretreatment of cells with recombinant SS^Ext^ mIFNγ(SP)_10_ could lead to the down-regulation of the *Influenza virus* replication.

### Secretion of SS^Ext^ mIFNγ(SP)_10_ Was Greatly Improved in 8-Day-Old *N. benthamiana* Cell Culture

**FIGURE 6:**
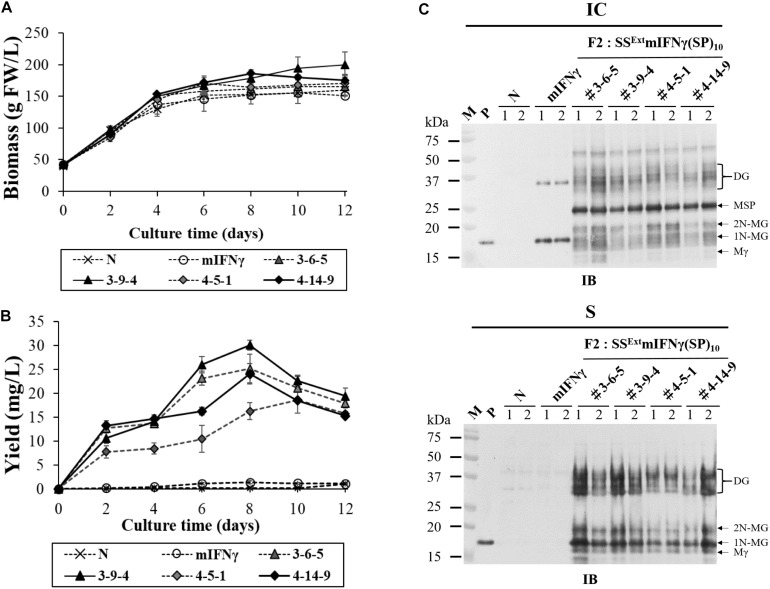
Secretion of SS^Ext^ mIFNγ(SP)_10_ in *N. benthamina* suspension cell culture. Suspension cells established from wilde-type (non-transgenic, N), transgenic *N. benthamina* line expressing mIFNγ (F2: 50-1-3), or four transgenic lines expressing SS^Ext^ mIFNγ(SP)_10_ (F2: 3-6-5/3-9-4/4-5-1/4-14-9) were cultured in 25 ml SH media (containing 100 mg/L kanamycin). **(A)** Time course of cell biomass accumulation. **(B)** The production of secreted proteins in culture media analyzed by ELISA. **(C)** Detection of protein accumulation in the intracellular (IC) or secreted (S) fractions, analyzed by western blot with mIFNγ-specific antibodies, after an 8-day culture period. Various forms of SS^Ext^ mIFNγ(SP)_10_ proteins were indicated on the right. M, marker; P, positive control, purified mIFNγ protein derived from *E. coli*; Mγ, M mIFNγ; 1N-MG monoglycosylated mIFNγ, 2N-MG diglycosylated mIFNγ; MSP, M unprocessed SS^Ext^ mIFNγ(SP)_10_; DG, D Glyco-mIFNγ.

Plant cell suspension cultures allow contained production of recombinant proteins under aseptic conditions (Santos et al., 2016), and are therefore suitable for the preparation of pharmaceutical proteins in compliance with cGMP regulations. In addition, the secretion of TPs into the culture medium would facilitate continuous production and greatly simplify the purification process. Thus, to verify whether SS^Ext^ mIFNγ(SP)_10_ produced by BaMV-based vector could be secreted into the medium, we established suspension cell lines using callus derived from transgenic *N. benthamiana* leaves expressing mIFNγ (F2: 50-1-3) and SS^Ext^ mIFNγ(SP)_10_ (F2: 3-6-5, 3-9-4, 4-5-1, 4-14-9), with non-transgenic cell line as a negative control (N). Each transgenic or non-transgenic cell line was cultured in SH medium to quantify cell biomass and secreted TP yield by ELISA at different culture intervals. The result of cell biomass quantification showed the transgenic and non-transgenic cell lines had similar growth curves, indicating that chimeric BaMV transgene did not affect cell growth (Figure 6A). The time course of TP secretion was further examined by ELISA as shown in Figure 6B. The result revealed that the yields of secreted TP were dramatically increased after two days of culture and continued increasing till the eighth day. The maximum yield of the secreted SS^Ext^ mIFNγ(SP)_10_ in 8-day-old *N. benthamiana* transgenic (F2: 3-9-4 line) cell culture was up to 30 ± 1 mg/L, which was 21 to 22 folds greater than the accumulation level of mIFNγ (F2: 50-1-3 line) control. The product of secreted SS^Ext^ mIFNγ(SP)_10_ in the 8-day-old culture was further verified by IB with mIFNγ-specific antibodies. As shown in Figure 6C, the result of IB showed that all four transgenic *N. benthamiana* cell lines expressing SS^Ext^ mIFNγ(SP)_10_ could post-translationally modify the secreted products, including Mγ (16 kDa), 1N-MG (18 kDa), 2N-MG (20 kDa) and D Glyco-mIFNγ (36–40 kDa), as opposed to that expressing mIFNγ (F2: 50-1-3 line) and non-transgenic cell line (Figure 6C, bottom panel). This observation is in agreement with the detection of secreted product in AWF of *N. benthamiana* leaves inoculated with the corresponding constructs. In contrast, large amounts of the unprocessed MSP (25 kDa, retaining SS^Ext^ and (SP)_10_ polypeptides) were detected in the IC fraction compared to other forms of TP (Figure 6C, upper panel). In addition, most of the expressed TP products from transgenic cell suspension cultures were secreted into the culture media (the S fraction) with 65–70% secretion efficiency (Supplementary Table 2). Together, these results demonstrated that the BaMV-based vector, coupled with suitable signal peptides, could be applied in the production of pharmaceutical proteins from the medium of *N. benthamiana* suspension cell cultures.

## Discussion

Solubility and secretion efficiency of the TP are among the major limiting factors for the downstream processing in the plant-based production systems. The post-translational modification and bioactivity of the TP are the key determinants for the applicability of the products. In this study, we developed a protein expression system with high potential for the large-scale production of plant-made TP, SS^Ext^ mIFNγ(SP)_10_. Based on the BaMV-derived vector, pKB19mIFNγ, that we developed previously (Jiang et al., 2019), the native SS^Ext^ from *N. benthamiana* and (SP)_10_ signal peptides were incorporated as the fusion tags of the TP, mIFNγ, as the apoplast leader sequence and secretion booster, to enhance the solubility and secretion of SS^Ext^ mIFNγ(SP)_10_. We showed that the fusion tags enabled the purification of target as the secreted glycoproteins from the soluble fraction of vacuum-infiltrated leaves and the medium of transgenic *N. benthamiana* suspension cell cultures. The yields of SS^Ext^ mIFNγ(SP)_10_ proteins reached 94 ± 7 mg/kg FW and 30 ± 1 mg/L culture media in vacuum-infiltrated leaves and suspension cell cultures, respectively. Additionally, the N-glycosylation profile and anti-virus activity of the soluble SS^Ext^ mIFNγ(SP)_10_ were verified in this study. The results indicated that this improved expression system may serve as an attractive alternative for the production of secreted, soluble, glycosylated, and bioactive PMPs.

### The Choice of Optimal SS for the Efficient Production of Secreted, Soluble, and Glycosylated Protein

Glycosylation and proteolytic maturation during the transportation through the secretory pathway are important co- or post-translational modifications of PMPs. One of the major aim of this study is to improve the secretion efficiency of mIFNγ by the fusion of optimal SSs derived from plants into the BaMV-based vector system. Therefore, various plant-derived SSs were taken into consideration to increase the yields of secreted mIFNγ *in N. benthamiana*, including a novel, native SS from the extensin of *N. benthamiana* cloned in this study. Our result showed that the yields of various SSmIFNγ varied widely, reaching a 28-fold difference between the highest (the native SS^Ext^) and lowest (SS^*Ramy*^) secretors (Figure 1C). It has been shown that the secretion efficiency of recombinant proteins are greatly influenced by the fusion of specific SS in various hosts, including CHO cells with a 100-fold difference (Kober et al., 2013), *Chlamydomonas reinhardtii* with a 10-fold difference (Molino et al., 2018) and *Oryza sativa* L. with a 2-fold difference (Huang et al., 2015). Our findings showed that SS^Ext^ led to an increase of yield up to 2.7 folds in IC fraction (Figure 1C), as compared to the negative control of mIFNγ without SS^Ext^. In addition, SS^Ext^ would facilitate the secretion of various forms of TP, including Mγ (16 kDa), 1N-MG (18 kDa), 2N-MG (20 kDa) and DG (36–41 kDa), in the extracellular (apoplast) space, which is not observed from the leaves infiltrated with the negative control construct of pKB19mIFNγ (Figures 1B,C). Thus, the results revealed that the *N. benthamiana* SS^Ext^ is a potent plant-derived SS for efficient production of secreted Glyco-mIFNγ. However, it is worth noting that different SSs might perform differently for different TPs, and it is necessary to identify the optimal SS for customization. The BaMV-based vectors with different SSs constructed in this study provide a convenient system for the screening of the optimal SS for different TPs for future studies.

The addition of HypGP signal, (SP)_10_, fused to C terminal of SS^Ext^ mIFNγ (designated SS^Ext^ mIFNγ(SP)_10_), was also shown to enhance the secretion of mIFNγ in either apoplast space of *N. benthamiana* leaf tissues (Figure 2B) or suspension cell cultures (Figures 6B,C). These results are in agreement with the previous studies for the production of high amount of secreted recombinant proteins in tobacco BY2 suspension cells (Xu et al., 2007, 2010; Zhang et al., 2019a), tobacco hairy root cultures (Zhang et al., 2019b) and green microalga *Chlamydomonas reinhardtii* (Ramos-Martinez et al., 2017). HypGP modules have been shown to be molecular carriers to boost the transport of fused proteins along secretion pathway into extracellular space (Zhang et al., 2019b). Interestingly, in our study, both the yield and solubility of SS^Ext^ mIFNγ(SP)_10_ were increased significantly, up to 6 and 2.7 folds, respectively, higher than those from the *N. benthamiana* leaf tissues expressing the control mIFNγ (Table 1 and Figure 2C). Thus, the results demonstrated that it is advantageous to apply HypGP technology in combination with species-specific SS^Ext^ in *N. benthamiana* to increase both the yield and solubility of prone-to-aggregate TP, which may further enhance the efficiency in the subsequent protein purification without the needs for protein refolding and re-solubilization processes. The mIFNγ protein has the tendency to self-associate into disordered aggregates through hydrophobic interactions (Jin et al., 2006; Razaghi et al., 2016). Thus, many scientists have explored different heterologous expression systems and used different strategies to enhance the production of soluble mIFNγ, such as fusing with artificial SS to transport TPs into periplasm in *E.coli* (BL-21) (Hernandez et al., 2008), or with solubility enhancing tags, e.g., small ubiquitin-like modifier (SUMO) in *E. coli* co-expressing two chaperone systems (dnaK–dnaJ–grpE and groES–groEL) (Tileva et al., 2016). However, the absence of proper post-translational modification in *E. coli* systems rendered the TP non-glycosylated, which reduced the half-life of mIFNγ in blood, usually with a lower biological activity (Hooker and James, 1998; Razaghi et al., 2016). The results of this study showed that the modified BaMV-based expression system allowed for the production of TPs with eukaryotic PTM and also overcame the aggregation nature of mIFNγ, increasing greatly the solubility of the protein product. The accumulation level of SS^Ext^ mIFNγ(SP)_10_ in the inoculated leaves reached up to 489 ± 28 mg/kg and 15.2 ± 0.98 mg/kg FW, corresponding to approximately 7.5% and 1.7% of TSP in IC and AWF fractions, respectively (Table 1), which are comparative or higher than those observed from previous studies for plant-made mIFNγ, including (1) rice suspension cells systems (131.6 ng/g cells biomass and 17.4 ng/mL media) (Chen et al., 2004) (2) tobacco leaves system (20 μg/g FW) (Wu et al., 2009); (3) a chimeric ZYMV-based vector system in *Chenopodium quinoa* leaves (1–1.2 mg/100 g FW) (Nassaj Hosseini et al., 2013); (4) transgenic tobacco chloroplast system (360 μg/g FW) (Leelavathi and Reddy, 2003); and (5) an extensin-like protein (ELP)-fusion system in tobacco leaves (244 ng/μg TSP) (Heidari-Japelaghi et al., 2019) and in transgenic tobacco plants (4% of TSP) (Heidari-Japelaghi et al., 2020).

### Post-Translational Modification During Protein Transportation Through the Secretion Pathway

Plants possess highly conserved secretory pathway similar to mammalian cells in terms of PTM (ex. glycosylation, phosphorylation, and proteolytic maturation), protein folding and assembly into biological active forms of therapeutic proteins (Walsh and Jefferis, 2006; Schneider et al., 2014). It has been reported that the glycosylation form of hIFNγ can profoundly affect both solubility and stability (Razaghi et al., 2016). The different eukaryotic expression systems producing specific N-glycan would also affect therapeutic applications. For example, mammalian-expressed hIFNγ with complex-type glycosylation exhibited higher levels of biological activity against cancer cells compared to its deglycosylated form (Razaghi et al., 2017b). However, the heterogeneous N-glycan side chains of plant-produced hIFNγ have not yet been completely identified to date. Thus, we further analyzed the glycan profiles of plant-made SS^Ext^ mIFNγ(SP)_10_ variants by using LC-MS/MS and PNGase A deglycosylation analysis. The results showed that 1N-MG (18 kDa), 2N-MG (20 kDa) and DG (36–40 kDa) were N-glycosylated at N^56^ and N^128^ (Figure 3), which corresponded to native mIFNγ glycosylation sites at N^25^ and N^97^, respectively (Sareneva et al., 1996; Razaghi et al., 2016). It also revealed that the approximately 96% of the N^56^ glycosylation site of SS^Ext^ mIFNγ(SP)_10_ was dominated by α-(1,3)-fucose complex-type glycan; whereas approximately 58% of N^128^ was occupied by considerably heterogeneous glycan (Figure 3D). The high N-glycan variations have also been observed in leukocytes expressing native hIFNγ (N^97^) (Sareneva et al., 1996; Razaghi et al., 2016), corresponding to the N^56^ of our plant-made SS^Ext^ mIFNγ(SP)_10_.Thus, the result indicated that the BaMV-based protein expression system in plants has the potential of producing TPs with glycosylation types similar to those of mammalian systems. In addition, a previous study has shown the glycosylation site at N^25^, corresponding to the N^56^ of our plant-made SS^Ext^ mIFNγ(SP)_10_, is the key determinant for efficient dimerization and secretion of IFNγ (Sareneva et al., 1994). Thus, although the N^128^ glycosylation site of SS^Ext^ mIFNγ(SP)_10_ produced in *N. benthamiana* was not completely glycosylated, the partially glycosylated protein (1N-MG) may still be eligible for secretion and dimerization with only one glycosylation at N^56^ (1N-MG).

In plant cells, as in other mammalian cells, the generation of oligosaccharide N-linked-Asn residues starts in the ER, followed by several maturation steps involving removal and the addition of sugar residues during ER transportation to Golgi complex (Faye et al., 2005; Gomord et al., 2010). In our case, the nascent mIFNγ was transported along the secretory pathway and undergoes several maturation steps to generate high-mannose-type N-glycan of mIFNγ in ER. Eventually, the addition of sugar (e.g., fucose, N-acetylglucosamine, xylose and galactose) yields relatively abundant complex-type structures of mIFNγ at N^56^ and N^128^ in Golgi complex. However, we hypothesized that most plant-derived SS^Ext^ and (SP)_10_ signals were trimmed by protease hydrolysis through the secretory pathways and the subsequent proteolytic maturation processes, based on the results of LC-MS/MS and deglycosylation analyses. These results indicated that the majority of SS^Ext^ mIFNγ(SP)_10_ were truncated to the root form of mIFNγ without SS^Ext^ signal and intact HypGP glycomodule, which may be particularly susceptible to posttranslational proteolysis at C-terminal end of hIFNγ as observed in CHO cell- (Curling et al., 1990) or *Spodoptera frugiperda* (Sf9) (Curling et al., 1990) cell-derived hIFNγ with C-terminus-cleaved form. Therefore, the truncated mIFNγ monomer (16 kDa) was glycosylated through post translational modification into the proteolytically maturated form, 1N-MG (18 kDa) and 2N-MG (20 kDa), which then underwent self-assembly into the biologically active form of DG (36–40 kDa). This PTM process is similar to that of α1-antichymotrypsin or α1-antitrypsin-(AP)_20_ fusion, which were also modified by glycosylation and proteolytic trimming in BY2 suspension cells (Benchabane et al., 2009; Zhang et al., 2019a), with remarkable resemblance to α1-antitrypsin-(AP)_20_ fusion which was also partially cleaved at the C-terminus to remove the (AP)_20_ HypGP signal during their transit through the secretory pathway (Zhang et al., 2019a).

### Comparison With Commercial Recombinant mIFNγ for Activity Against Viruses

Commercial recombinant mIFNγ (ACTIMMUNE^®^, IFNγ-1b) has been used as a clinically effective drug for severe malignant osteopetrosis (Key et al., 1992) and chronic granulomatous disease (Bolinger and Taeubel, 1992) after being approved by the U.S. Food and Drug Administration (FDA). Recombinant human mIFNγ has been studied for therapeutic applications against a variety of viruses, like *hepatitis B virus* (Parvez et al., 2006), *Dengue virus* (Chen et al., 2004), *Vesicular stomatitis virus* (VSV) (Jin et al., 2006), and *Encephalomyocarditis virus* (EMCV) (Leelavathi and Reddy, 2003; Wang et al., 2014). The biological activity assays in this study showed that plant-made SS^Ext^ mIFNγ(SP)_10_ could induce the anti-viral state to chimeric SINV-eGFP infected cells and inhibit its proliferation with a specific activity of 3 × 10^7^ IU/mg protein, which is higher than that of the commercial P-IFNγ, 2 × 10^7^ IU/mg protein (Figures 4A–C). Also, SS^Ext^ mIFNγ(SP)_10_ could inhibit IVA replication as determined by the reduction of both NS1 and NP protein expression, suggesting that SS^Ext^ mIFNγ(SP)_10_ has the biological properties against different viruses similar to the commercial PC-IFNγ.

### The Advantages of Producing Secreted Proteins Using Plant-Suspension Cells

The productions of plant-made pharmaceuticals are strictly regulated by different government agencies in different countries, which usually require the compliance with the current Good Manufacturing Practice (cGMP). For efficient production of secreted TP in conformity with the cGMP regulations, we established suspension cell lines of transgenic *N. benthamiana* expressing SS^Ext^ mIFNγ(SP)_10_ that could produce secretory forms of Mγ (16 kDa), 1N-MG (18 kDa), 2N-MG (20 kDa) and DG (36–40 kDa) from SH culture medium (Figure 6C). The result of ELISA showed that *N. benthamiana* transgenic cell line F2: 3-9-14 produced the highest amount of TP, up to 30 ± 1 mg/L, which was 21 to 22 folds greater than the expression of mIFNγ control (Figure 6B). The improved production of secreted protein was in agreement with previous studies for BY2 cell systems based on HypGP technology expressing other PMPs, such as IFNα2-(SP)_10_ (17–28 mg/L culture medium) (Xu et al., 2007), hGH-(SP)_10_ (16–35 mg/L culture media) (Xu et al., 2010), and AAT-(AP)_20_ (34.7 mg/L culture medium) (Zhang et al., 2019a). Other than yielding a higher amount of secreted TP, plant suspension cell culture systems also provide several advantages over the mammalian cell culture or whole plant systems, such as the lower risk of human pathogen contamination, less expensive culture medium, higher reproducibility and easier control for product quality by applying cGMP, making it a suitable alternative to achieve large-scale and safe production of PMPs (Hellwig et al., 2004; Muthamilselvan et al., 2016; Sukenik et al., 2018). Further optimization of the culture conditions for the transgenic *N. benthamiana* suspension cell cultures expressing SS^Ext^ mIFNγ(SP)_10_ is currently underway for the safe and large-scale production of TPs.

## Conclusion

To our knowledge, this is first report describing the efficient production of a secreted, soluble and glycosylated PMP, SS^Ext^ mIFNγ(SP)_10_, in *N. benthamiana* using a BaMV-based vector, with the incorporation of a novel, native SS^Ext^ and a (SP)_10_ designer tag. Our study also revealed that SS^Ext^ mIFNγ(SP)_10_ produced in the plant system could be properly processed by post-translational modification, including trimming of signal sequences and glycosylation, resulting in protein products that exhibited the biological activity to suppress SINV and IAV replication. Thus, this improved BaMV secretory expression system represented a worthy option for high-level expression of glycosylated and biologically active PMPs. Furthermore, the suspension cell culture systems established in this study provide valuable resources for further development of industrial-scale production of therapeutic proteins in compliance with cGMP regulations.

## Data Availability Statement

The original contributions presented in the study are included in the article/Supplementary Material, further inquiries can be directed to the corresponding author.

## Author Contributions

M-CJ, C-CH, W-LH, T-LH, N-SL, and Y-HH designed the study and analyzed the interpreted data. M-CJ, W-LH, and T-LH acquired the experimental data. M-CJ, C-CH, W-LH, T-LH, and Y-HH drafted and revised the manuscript. All authors contributed to the article and approved the submitted version.

## Conflict of Interest

The authors declare that the research was conducted in the absence of any commercial or financial relationships that could be construed as a potential conflict of interest.

## Supplementary Material

Supplementary Figure 1 Alignment of signal peptide sequences of three extensin proteins from *Nicotiana spp*.

Supplementary Figure 2 Effect of different surfactants on mIFNγER solubility.

Supplementary Figure 3 The purification of SS^Ext^ mIFNγ(SP)_10_ from vacuum-infiltrated leaves through acetic acid precipitation coupled with Ni^2+^-NTA and gel filtration chromatography.

Supplementary Figure 4 The purification of SS^Ext^ mIFNγ(SP)_10_ from vacuum-infiltrated leaves by gel filtration chromatography.

Supplementary Figures 5–14 LC-MS/MS analysis of the glycopeptides and glycan generated from SS^Ext^ mIFNg(SP)_10_.

Supplementary Figure 15 Detection of SS^Ext^ mIFNγ(SP)_10_ activity against SINV in SEK293-T cell.

Supplementary Table 1 The list of primers.

Supplementary Table 2 Secretion efficiency of TP in 8-day-old *N. benthamiana* cell culture.

## Abbreviations

AWF: apoplast washing fluid
BaMV: *Bamboo mosaic virus*
CaMV: *Cauliflower mosaic virus*
CBS: Coomassie Blue stain
cGMP: current Good Manufacturing Practice
CP: coat protein
D: dimeric
DG: dimeric glycosylated mIFN γ
DPI: days post-infiltration
DSP: during downstream purification
FW: fresh weight
Glyco-mIFN γ: glycosylated mIFN γ
HypGPs: hydroxyproline (Hyp)-O-glycosylated peptides
IAV: *Influenza A virus*
IB: immunoblotting
IC: intracellular
IFN γ: interferon gamma
LC-MS/MS: Liquid chromatography-tandem mass spectrometry
M γ: monomeric mIFN γ
M: monomeric
MG: monomeric glycosylated mIFN γ
mIFN γ: mature interferon gamma
*M*_*r*_: molecular weights
P1: pellet fraction after centrifugation at 1,000 × *g*
P30: pellet fraction after centrifugation at 30,000 × *g*
PAS: periodic acid-Schiff stain
PMPs: plant made-pharmaceuticals
PNGase A: Peptide-N-Glycosidase A
PNGase F: Peptide-N-Glycosidase F
PTM: post-translational modification
RuBisCO: Ribulose-1,5-bisphosphate carboxylase/oxygenase
S1: soluble fraction after centrifugation at 1,000 × *g*
S30: soluble fraction after centrifugation at 30,000 × *g*
SINV: *Sindbis virus*
SS(s): secretory signal(s)
TP(s): target protein(s)
TSP: total soluble protein.
