# Supplementary material for: Fusion of a Novel Native Signal Peptide Enhanced the Secretion and Solubility of Bioactive Human Interferon Gamma Glycoproteins in Nicotiana benthamiana Using the Bamboo Mosaic Virus-Based Expression System
Source: Front Plant Sci. 2020 Nov 12;11:594758. doi: 10.3389/fpls.2020.594758 (PMC7688984; doi:10.3389/fpls.2020.594758)
Supplement: Supplementary Figure 1 — Alignment of signal peptide sequences of three extensin proteins from Nicotiana spp. [file Table_1.pdf]

Supplementary Material

Supplementary Table 1 | The list of primers.

Primer	Sequence (5'→3')
F-*Mlu*I-*Nco*I-mIFNγ	GCACGCGTCCATGG[1]GCTGTTACTGCCAGGACC
R-*Not*I-TGA-His-mIFNγ	CGGCGGCCGC[1]TCAGTGGTGGTGGTGGTG
F-*Mlu*I-Ext	GCACGCGTATGGGGAAAATGGCTTCTCTATTTGCC**ACTCTTCTAGTAGTTTTAGTGTCA**[2]
R-*Mlu*I-Ext	CGACGCGTTGCTGAGCTTTCAGAAGCTAAGCTAAG**TGACACTAAAACTACTAGAAGAGT**[2]
F-*Mlu*I-αRAmy	GCACGCGTACCATGGGTAAGAACACCAGCAGCTTGTGTTTG**CTGCTCCTCGTGGTGCTC**[2]
R-*Mlu*I-αRAmy	GCACGCGTTGCTTGACCCGAGTTACAGGTCAAGCTGCA**GAGCACCACGAGGAGCAG**[2]
F-*Mlu*I-Pr1	GCACGCGTACCATGGGTTTGCCCTCATTTCTTTTGGTC**TCCACGTTGCTTCTTTTC**[2]
R-*Mlu*I-Pr1	GCACGCGTAGCCCTGCAGGAATGTGAAATTACAAG**GAAAAGAAGCAACGTGGA**[2]
F-*Mlu*I-VspA	GCACGCGTACCATGGGTAAAATGAAGGTCCTTGTTTTC**TTCGTTGCTACAATTTTG**
R-*Mlu*I-VspA	GCACGCGTAGCATGGCATTGCCATGCTAC**CAAAATTGTAGCAACGAA**[2]
F-*Mlu*I-PDI	GCACGCGTACCATGGCTAAAAACGTTAGCATTTTTGGTTTG**TTGTTTTCTCTTCTTGCT**[2]
R-*Mlu*I-PDI	GCACGCGTAGCGAAGATCTGAGAAGGAACCAA**AGCAAGAAGAGAAAACAA**[2]
F-SP-*Spe*I	GCACTAGT[1]TCACCCTCTCCAAGCCCTTCCCCA**TCGCCTAGTCCCTCACCATCC**
R-SP-*Spe*I	CGACTAGT[1]TGGGCTGGGAGAAGG**GGATGGTGAGGGACTAGGCGA**[2]

[1] Underlined nucleotides represent the restriction enzyme recognition sites.

[2] Nucleotides in bold indicate primer-overlapping regions.

Supplementary Table 2 | Secretion efficiency of TP in 8-day-old *N. benthamiana* cell culture.

TP[†]	Yield in media (mg/L)	Yield in IC[‡] (mg/ g FW)[§]	Cell biomass (g FW/L)[§]	% of Secreted products
nontransgenic	-	-	147-159	-
mIFNγ	0.4-1.4	0.046-0.068	146-164	3-11
SS^{Ext}mIFNγ$(SP)_{10}$	24-31	0.064-0.091	162-186	65-70

Yields were determined by ELISA from four transgenic lines expressing SS^{Ext}mIFNγ$(SP)_{10}$ (F2: 3-6-5/3-9-4/4-5-1/4-14-9) after an 8-day culture period. Abbreviations: [†] TP, target protein; [‡] IC, Intercellular space; [§] FW, fresh weight.

Supplementary Figures

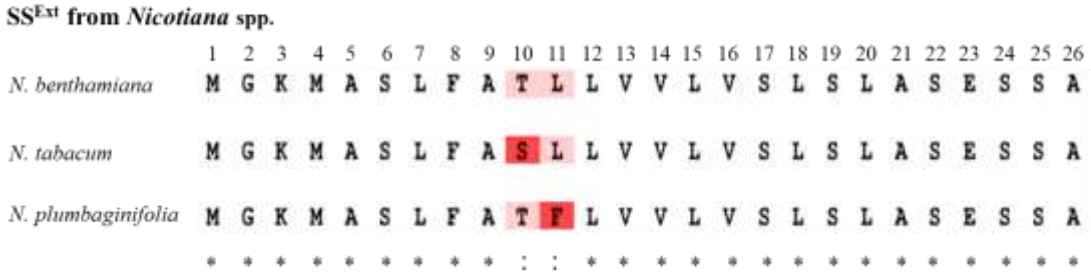

Supplementary Figure 1 | Alignment of signal peptide sequences of three extensin proteins from *Nicotiana spp*. The amino acid sequences of the extensin signal peptides from *N. tabacum*, *N. benthamiana*, and *N. plumbaginifolia* were aligned using ClustalW. The amino acids that are different in one of the three sequences were highlighted with a red background, whereas those conserved in the rest of the two sequences were indicated with a pink background. The consensus sequence is shown at the bottom of the alignment. Positions of identical or consensus residues were indicated by “ * ” or “ : ” signs, respectively.

A

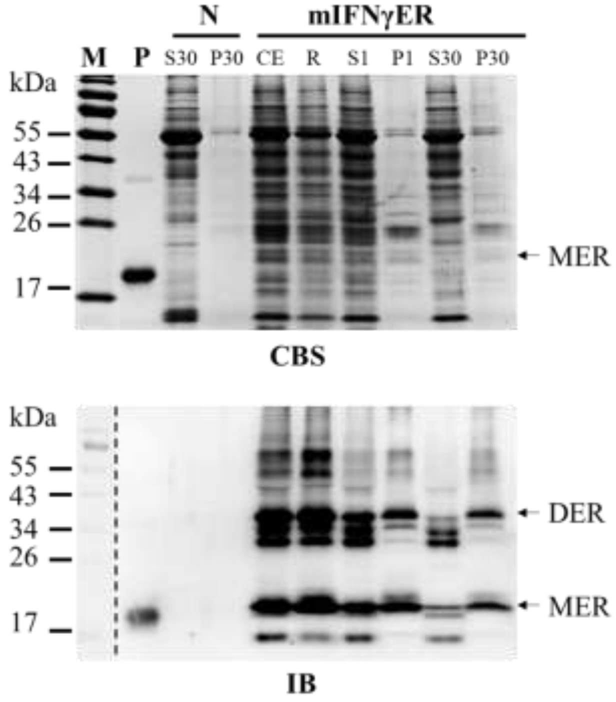
N
mIFNγER
M
P
S30
P30
CE
R
S1
P1
S30
P30
kDa
55
43
34
26
17
MER
CBS
kDa
55
43
34
26
17
DER
MER
IB

B

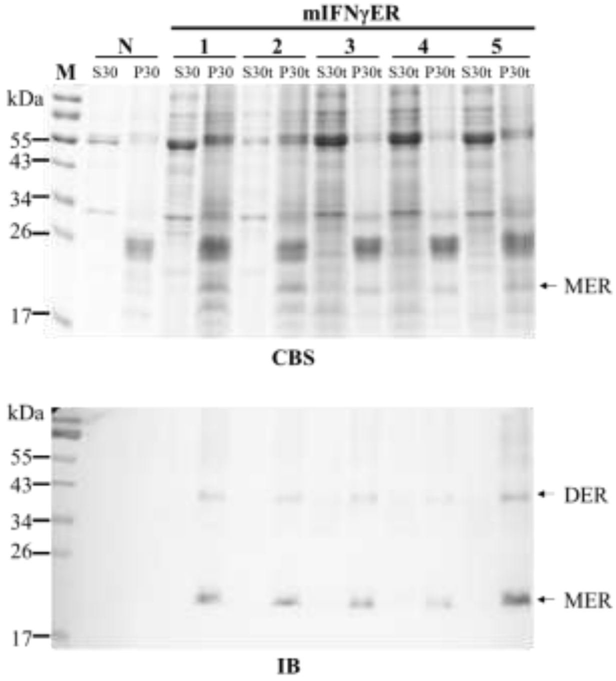
mIFNγER
N
1
2
3
4
5
M
S30
P30
S30
P30
S30t
P30t
S30t
P30t
S30t
P30t
S30t
P30t
kDa
55
43
34
26
17
MER
CBS
kDa
55
43
34
26
17
DER
MER
IB

Supplementary Figure 2 | Effect of different surfactants on mIFNγER solubility. *N. benthamiana* leaves were infiltrated with *A. tumefaciens* harboring plasmid pKB19mIFNγER as described in our previous study. The expressed proteins were extracted at 5 DPI with various surfactants to examine their effect on the solubility of mIFNγER. **(A)** The subcellular fractionation of mIFNγER in *N. benthamiana* leaves. The infiltrated leaves (20g) were extracted with buffer A (without SDS detergent) and then fractionated into crude extract (CE), leaf-residues (R), soluble fraction (S1), cell wall (P1), soluble cytosolic (S30) and membrane fractions (P30). M, marker; P, purified mIFNγ protein derived from *E. coli* (100 ng in CBS and 20 ng in IB) as positive control; N, healthy leaf as a negative control. MER, monomeric mIFNγER; DER, dimeric mIFNγER. **(B)** The solubilization of mIFNγER in P30 fraction. The isolated P30 were further treated with 1 M KCl (lane 2), 2% (v/v) Triton™ X-100 (lane 3), 2% (v/v) IGEPAL® CA-630 (lane 4), or 2% (v/v) Tween® 20 (lane 5) for mIFNγER solubilization, followed by centrifugation at 30,000 × *g* to yield the supernatant (S30t) and pellet (P30t) fractions. The samples were subsequently analyzed by SDS-PAGE, followed by staining with CBS and IB analysis with mIFNγ-specific antibodies.

.

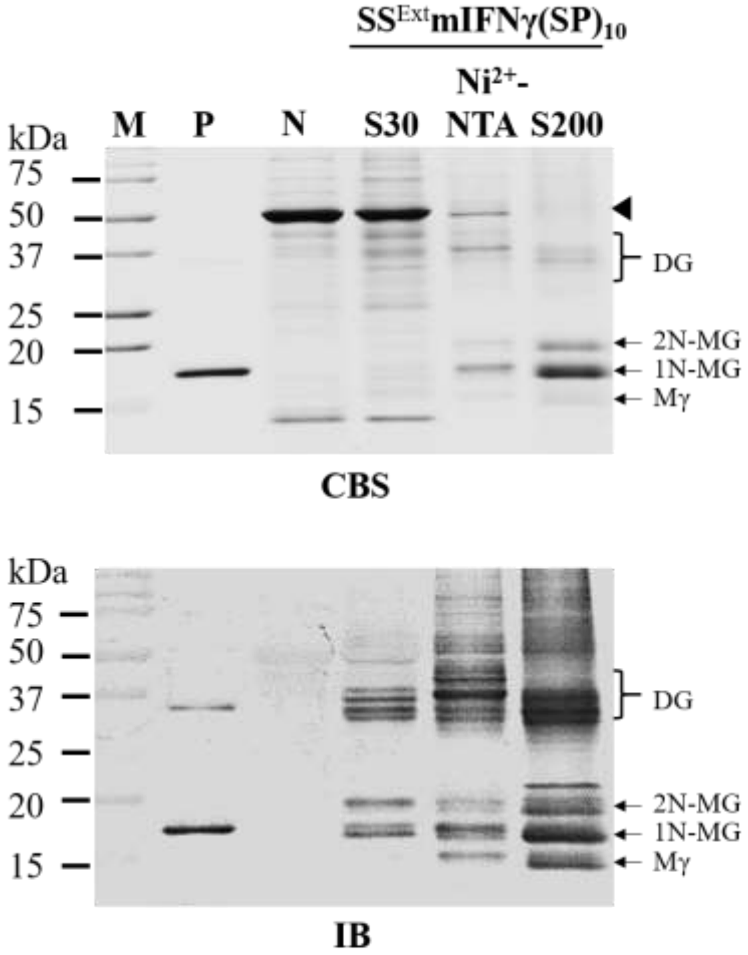

SSExtmIFNγ(SP)10
kDa
M
P
N
S30
Ni2+-
NTA
S200
75
50
37
25
20
15
DG
2N-MG
1N-MG
Mγ
CBS
kDa
75
50
37
25
20
15
DG
2N-MG
1N-MG
Mγ
IB

Supplementary Figure 3 | The purification of $SS^{Ext}mIFN\gamma(SP)_{10}$ from vacuum-infiltrated leaves through acetic acid precipitation coupled with Ni^{2+}-NTA and gel filtration chromatography. The S30 fraction was treated with acetic acid (pH 5.1) to precipitate RuBisCO proteins, indicated by the arrowhead (◀), and further purified through Ni^{2+}-NTA and gel filtration column (Superdex™ 200 pg, S200). Each collected fraction was subjected to SDS-PAGE analysis. The proteins in the gels or on the blots were visualized using CBS or IB, respectively. N, healthy leaf extract as a negative control. P, positive control, purified mIFNγ protein derived from *E. coli*. Various forms of $SS^{Ext}mIFN\gamma(SP)_{10}$ proteins were indicated on the right. Mγ, M mIFNγ; 1N-MG, M monoglycosylated mIFNγ; 2N-MG, M diglycosylated mIFNγ; DG, D Glyco-mIFNγ.

A

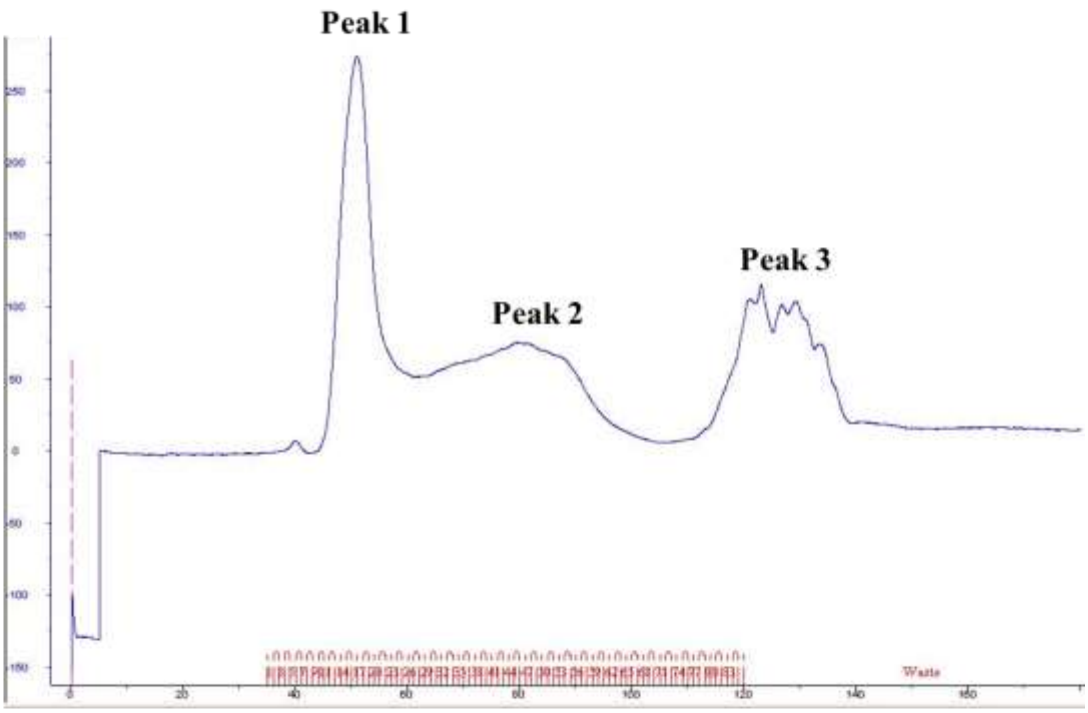
Peak 1
Peak 2
Peak 3
250
200
150
100
50
0
-50
-100
-150
0
20
40
60
80
100
120
140
160
Waste

B

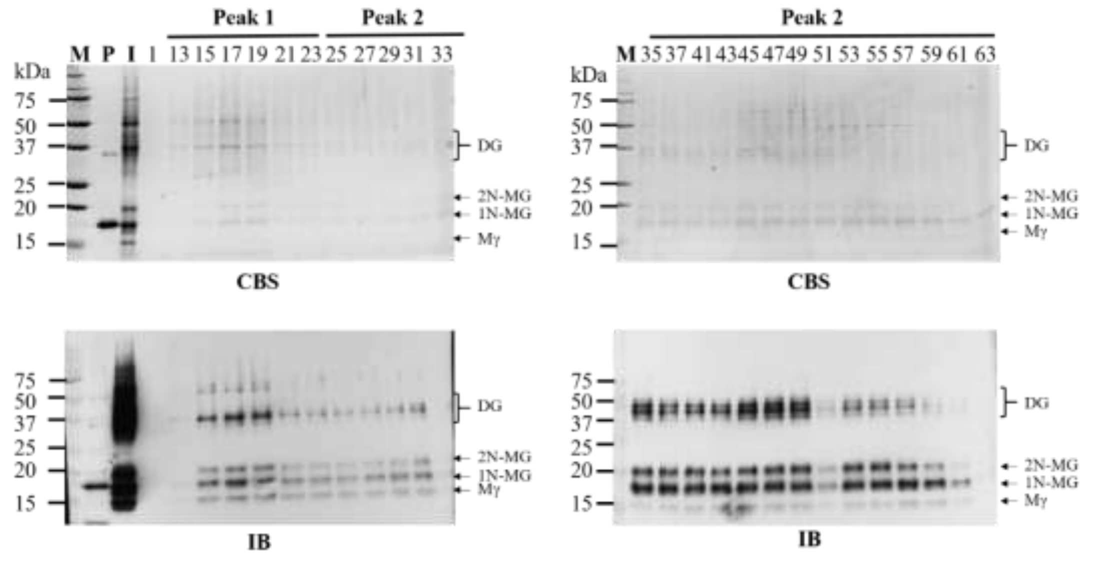
Peak 1
Peak 2
M P I 1 13 15 17 19 21 23 25 27 29 31 33
kDa
75
50
37
25
20
15
DG
2N-MG
1N-MG
Mγ
CBS
IB
Peak 2
M 35 37 41 43 45 47 49 51 53 55 57 59 61 63

Supplementary Figure 4 | The purification of $SS^{Ext}mIFN\gamma(SP)_{10}$ from vacuum-infiltrated leaves by gel filtration chromatography. The $SS^{Ext}mIFN\gamma(SP)_{10}$ fraction obtained from Ni^{2+}-NTA column chromatography was loaded onto a HiLoadTm 16/60 Superdex™ 200 pg (S200) column. Each 1-ml fraction was further obtained by a fast protein liquid chromatography (FPLC) system (AKTA purifier GE Health care systems). **(A)** The elution profiles as detected by UV 280. **(B)** The harvested protein fractions were analyzed by SDS-PAGE, followed by staining with CBS and IB analysis with mIFNγ-specific antibodies. M, Marker; I, injection samples; Peak 1, fraction number from 13 to 23; Peak 2, fraction number from 25 to 63. Mγ, M mIFNγ; 1N-MG, M monoglycosylated mIFNγ; 2N-MG, M diglycosylated mIFNγ; DG, D Glyco-mIFNγ.

Figure S5

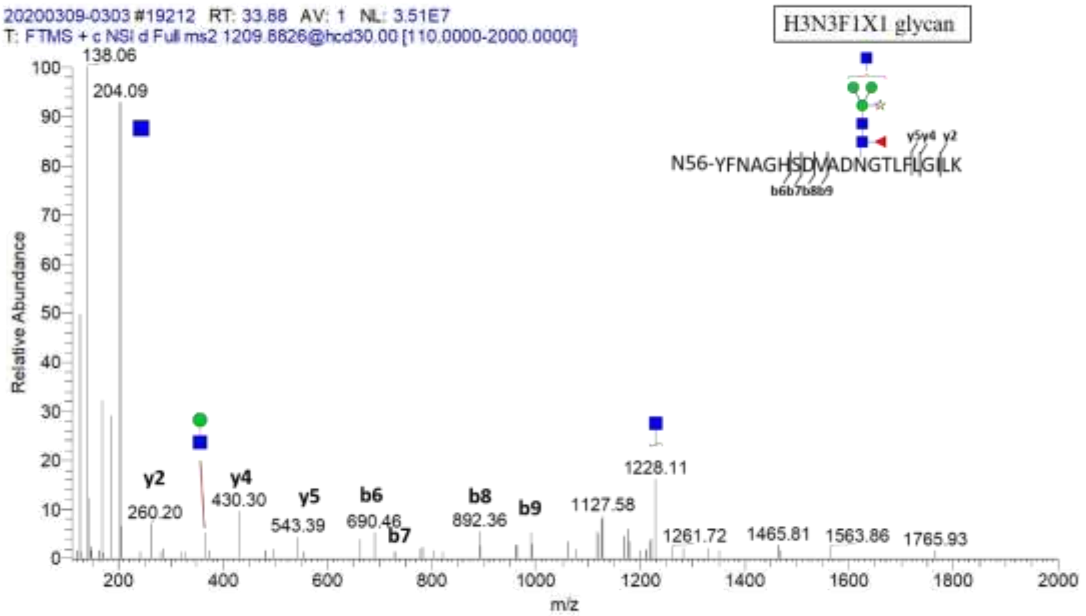

20200309-0303 #19212 RT: 33.88 AV: 1 NL: 3.51E7
T: FTMS + c NSI d Full ms2 1209.8826@hcd30.00 [110.0000-2000.0000]
H3N3F1X1 glycan
N56-YFNAGHSDVADNGTLFLGILK
y5y4 y2
b6b7b8b9
Relative Abundance
m/z
138.06
204.09
y2
260.20
y4
430.30
y5
543.39
b6
690.46
b7
b8
892.36
b9
1127.58
1228.11
1261.72
1465.81
1563.86
1765.93

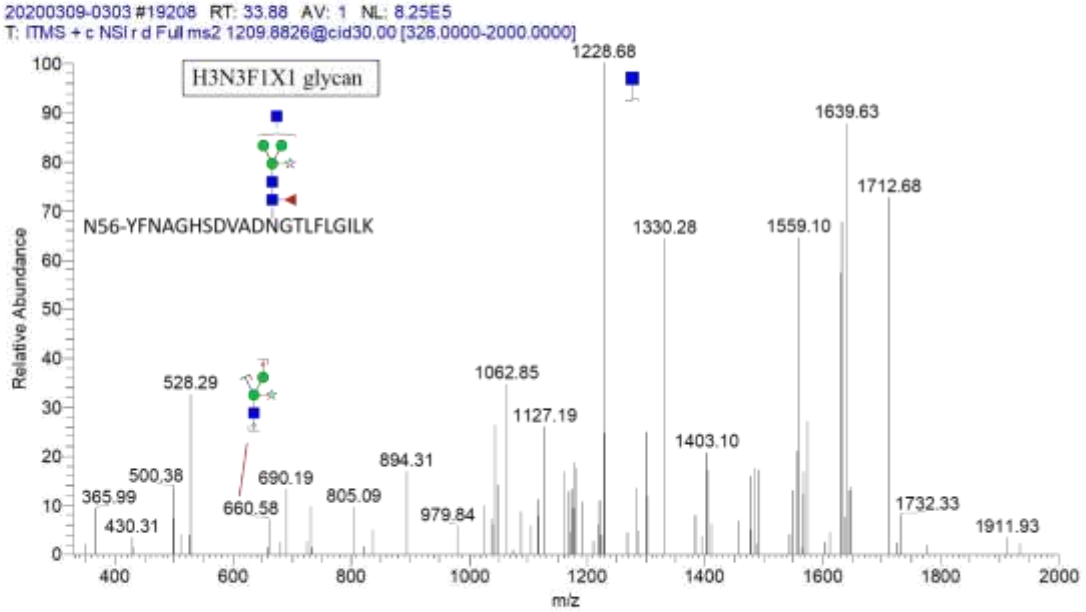

20200309-0303 #19208 RT: 33.88 AV: 1 NL: 8.25E5
T: ITMS + c NSI r d Full ms2 1209.8826@cid30.00 [328.0000-2000.0000]
H3N3F1X1 glycan
N56-YFNAGHSDVADNGTLFLGILK
Relative Abundance
m/z
1228.68
1639.63
1712.68
1330.28
1559.10
528.29
1062.85
1127.19
1403.10
500.38
365.99
430.31
660.58
690.19
805.09
894.31
979.84
1732.33
1911.93

Figure S6

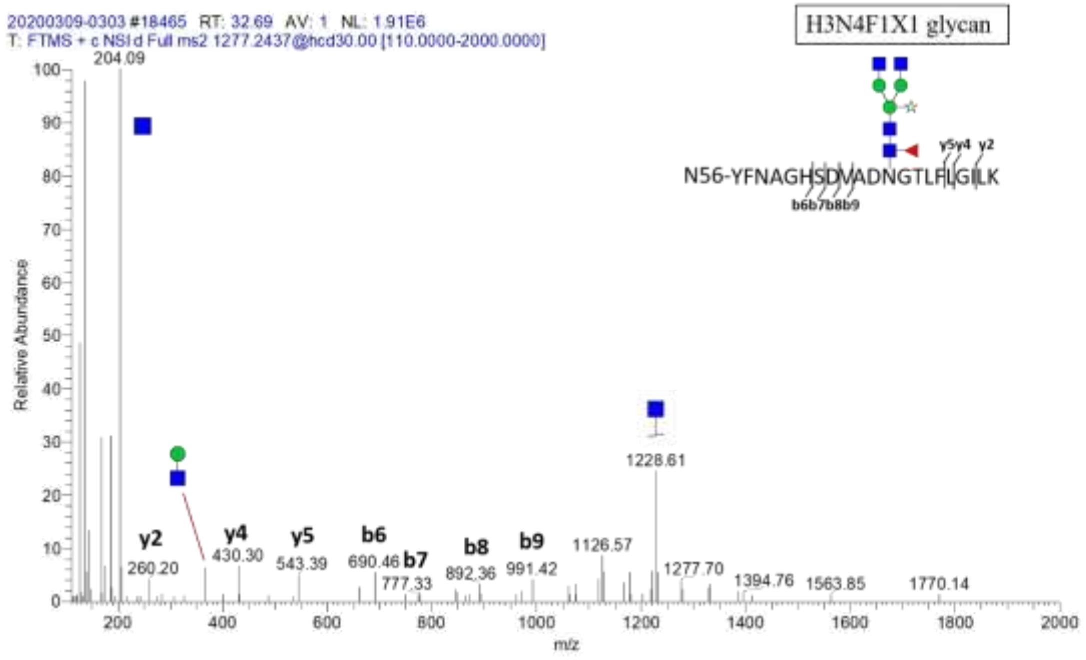
20200309-0303 #18465 RT: 32.69 AV: 1 NL: 1.91E6
T: FTMS + c NSI d Full ms2 1277.2437@hcd30.00 [110.0000-2000.0000]
H3N4F1X1 glycan
N56-YFNAGHSDVADNGTLFLGILK
y5y4 y2
b6b7b8b9
Relative Abundance
m/z
204.09
y2 260.20
y4 430.30
y5 543.39
b6 690.46
b7 777.33
b8 892.36
b9 991.42
1126.57
1228.61
1277.70
1394.76
1563.85
1770.14

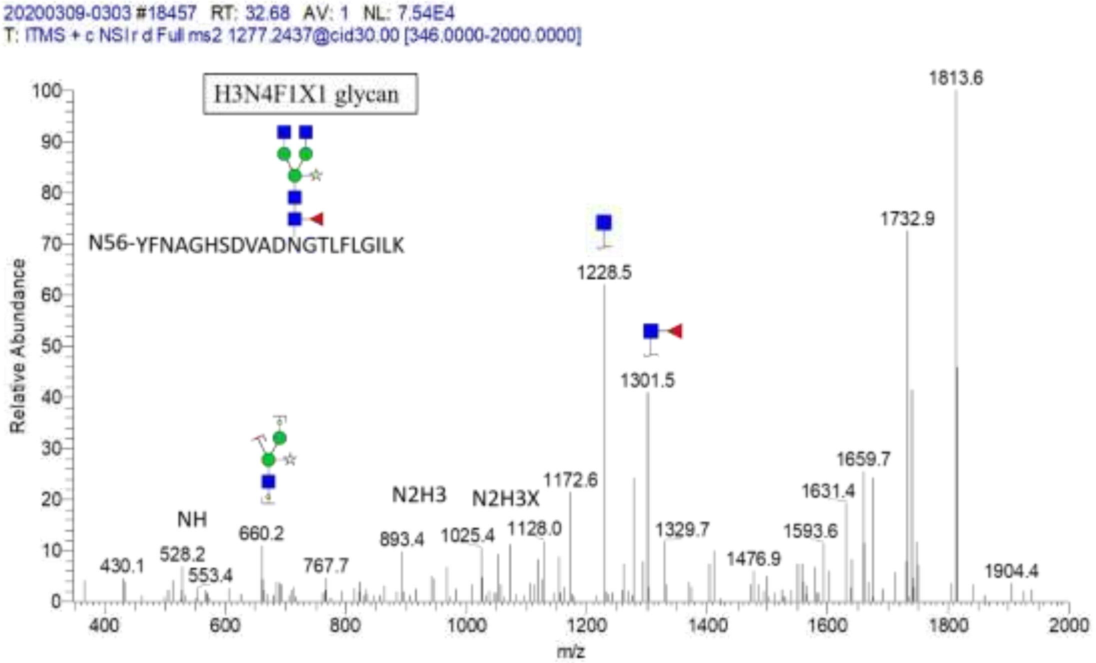
20200309-0303 #18457 RT: 32.68 AV: 1 NL: 7.54E4
T: ITMS + c NSI r d Full ms2 1277.2437@cid30.00 [346.0000-2000.0000]
H3N4F1X1 glycan
N56-YFNAGHSDVADNGTLFLGILK
Relative Abundance
m/z
430.1
NH
528.2
553.4
660.2
767.7
N2H3
893.4
N2H3X
1025.4
1128.0
1172.6
1228.5
1301.5
1329.7
1476.9
1593.6
1631.4
1659.7
1732.9
1813.6
1904.4

Figure S7

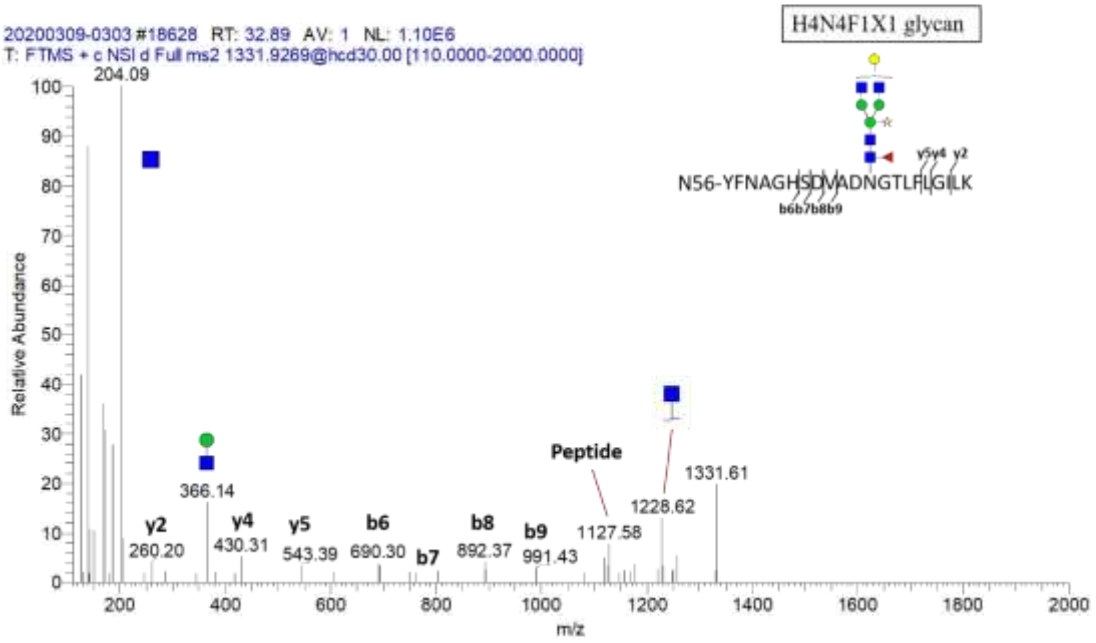

20200309-0303 #18628 RT: 32.89 AV: 1 NL: 1.10E6
T: FTMS + c NSI d Full ms2 1331.9269@hcd30.00 [110.0000-2000.0000]
H4N4F1X1 glycan
N56-YFNAGHSDVADNGTLFLGILK
Relative Abundance
m/z
204.09
366.14
y2 260.20
y4 430.31
y5 543.39
b6 690.30
b7
b8 892.37
b9 991.43
Peptide
1127.58
1228.62
1331.61

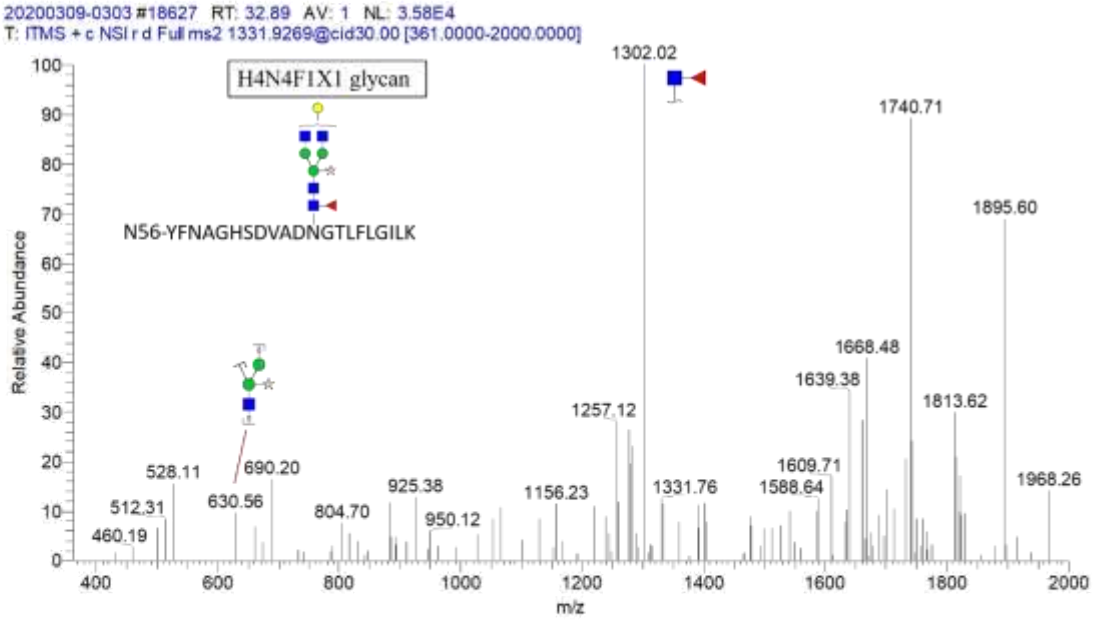

20200309-0303 #18627 RT: 32.89 AV: 1 NL: 3.58E4
T: ITMS + c NSI r d Full ms2 1331.9269@cid30.00 [361.0000-2000.0000]
H4N4F1X1 glycan
N56-YFNAGHSDVADNGTLFLGILK
Relative Abundance
m/z
1302.02
1740.71
1895.60
1668.48
1639.38
1813.62
1257.12
528.11
690.20
512.31
630.56
460.19
804.70
925.38
950.12
1156.23
1331.76
1609.71
1588.64
1968.26

Figure S8

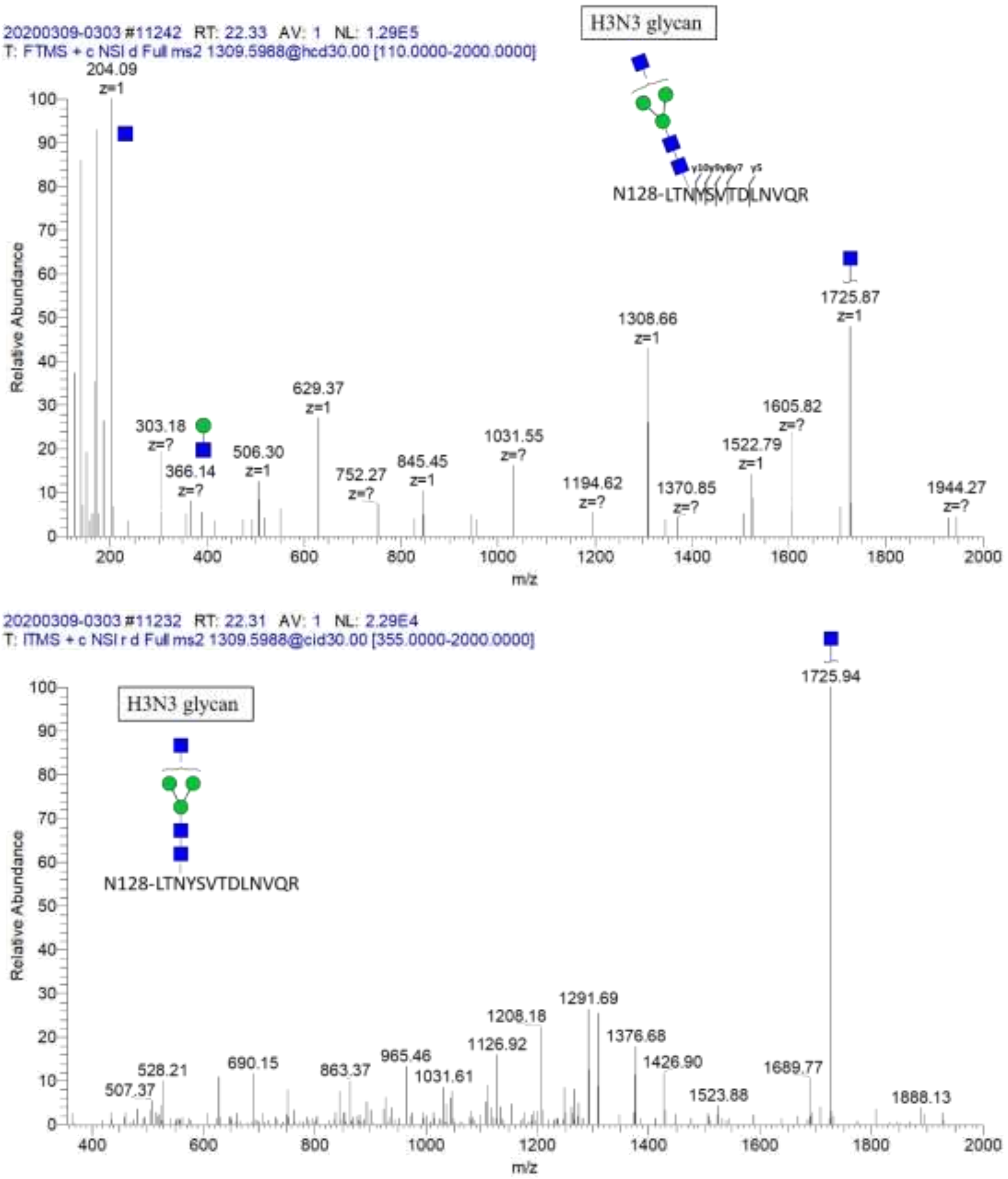
20200309-0303 #11242 RT: 22.33 AV: 1 NL: 1.29E5
T: FTMS + c NSI d Full ms2 1309.5988@hcd30.00 [110.0000-2000.0000]
H3N3 glycan
N128-LTNYSVTDLNVQR
204.09 z=1
303.18 z=?
366.14 z=?
506.30 z=1
629.37 z=1
752.27 z=?
845.45 z=1
1031.55 z=?
1194.62 z=?
1308.66 z=1
1370.85 z=?
1522.79 z=1
1605.82 z=?
1725.87 z=1
1944.27 z=?
Relative Abundance
m/z
20200309-0303 #11232 RT: 22.31 AV: 1 NL: 2.29E4
T: ITMS + c NSI r d Full ms2 1309.5988@cid30.00 [355.0000-2000.0000]
H3N3 glycan
N128-LTNYSVTDLNVQR
507.37
528.21
690.15
863.37
965.46
1031.61
1126.92
1208.18
1291.69
1376.68
1426.90
1523.88
1689.77
1725.94
1888.13
Relative Abundance
m/z

Figure S9

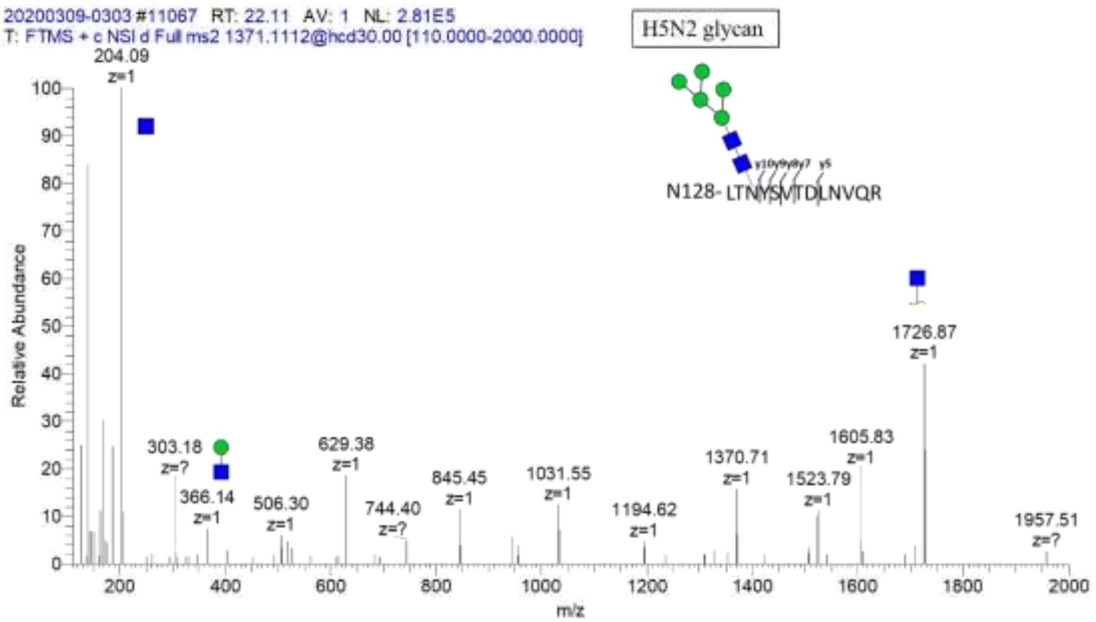
20200309-0303 #11067 RT: 22.11 AV: 1 NL: 2.81E5
T: FTMS + c NSI d Full ms2 1371.1112@hcd30.00 [110.0000-2000.0000]
H5N2 glycan
N128- LTNYSVTDLNVQR
Relative Abundance
m/z
204.09 z=1
303.18 z=?
366.14 z=1
506.30 z=1
629.38 z=1
744.40 z=?
845.45 z=1
1031.55 z=1
1194.62 z=1
1370.71 z=1
1523.79 z=1
1605.83 z=1
1726.87 z=1
1957.51 z=?

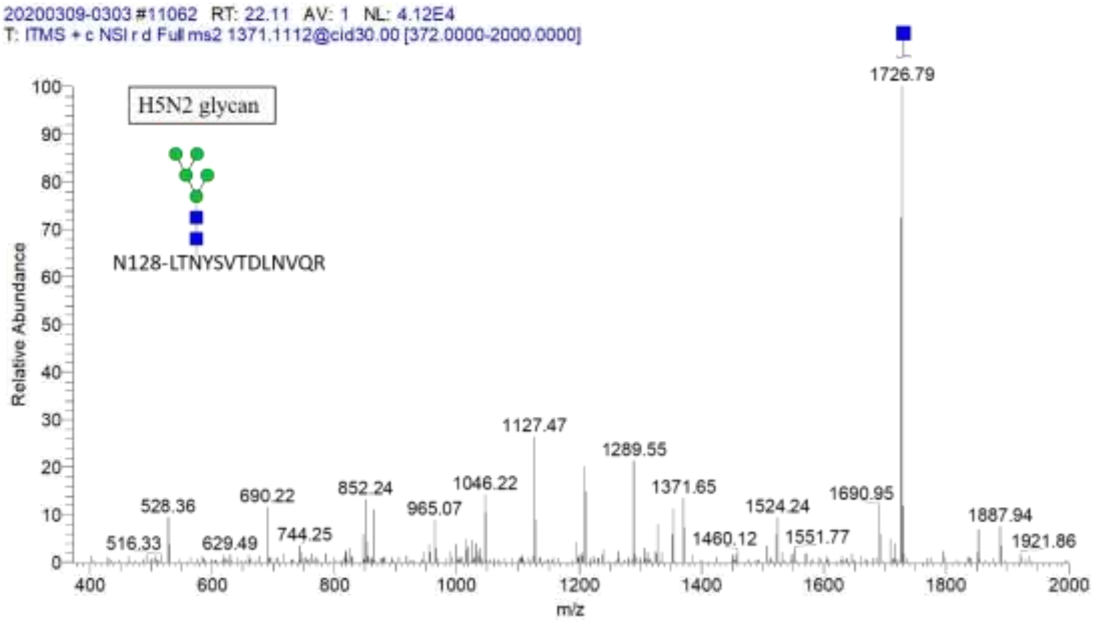
20200309-0303 #11062 RT: 22.11 AV: 1 NL: 4.12E4
T: ITMS + c NSI r d Full ms2 1371.1112@cid30.00 [372.0000-2000.0000]
H5N2 glycan
N128-LTNYSVTDLNVQR
Relative Abundance
m/z
516.33
528.36
629.49
690.22
744.25
852.24
965.07
1046.22
1127.47
1289.55
1371.65
1460.12
1524.24
1551.77
1690.95
1726.79
1887.94
1921.86

Figure S10

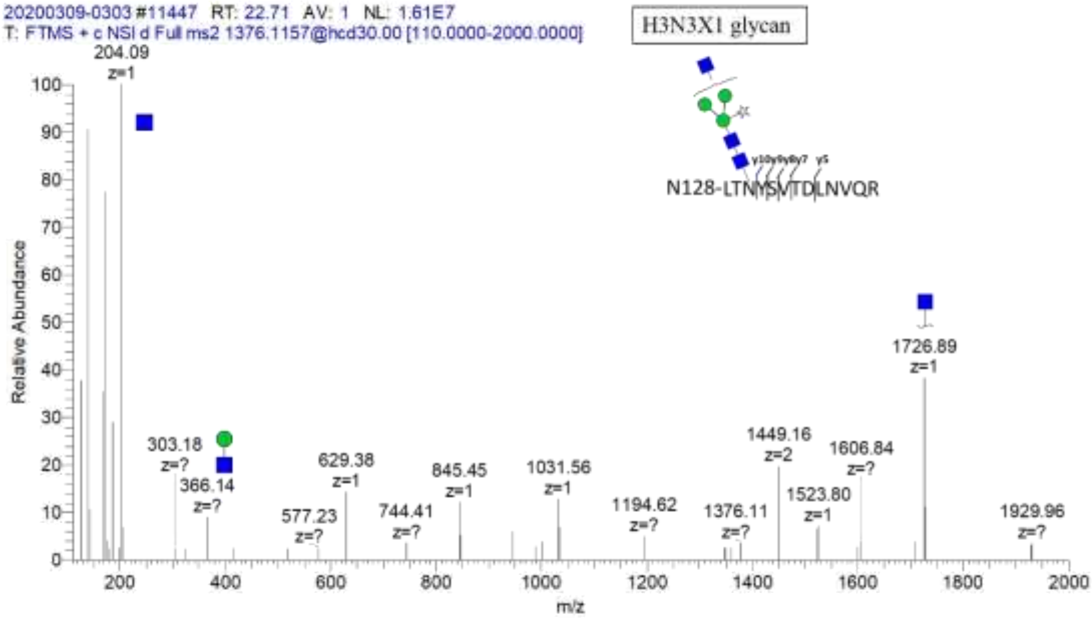

20200309-0303 #11447 RT: 22.71 AV: 1 NL: 1.61E7
T: FTMS + c NSI d Full ms2 1376.1157@hcd30.00 [110.0000-2000.0000]
H3N3X1 glycan
N128-LTNYSVTDLNVQR
204.09 z=1
303.18 z=?
366.14 z=?
577.23 z=?
629.38 z=1
744.41 z=?
845.45 z=1
1031.56 z=1
1194.62 z=?
1376.11 z=?
1449.16 z=2
1523.80 z=1
1606.84 z=?
1726.89 z=1
1929.96 z=?
Relative Abundance
m/z

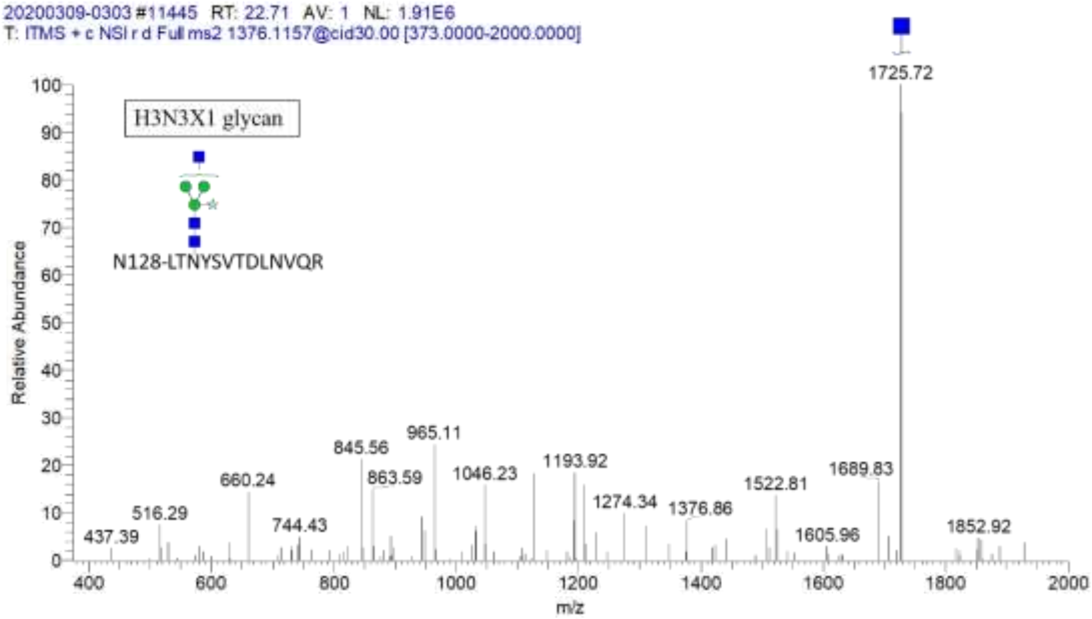

20200309-0303 #11445 RT: 22.71 AV: 1 NL: 1.91E6
T: ITMS + c NSI r d Full ms2 1376.1157@cid30.00 [373.0000-2000.0000]
H3N3X1 glycan
N128-LTNYSVTDLNVQR
1725.72
437.39
516.29
660.24
744.43
845.56
863.59
965.11
1046.23
1193.92
1274.34
1376.86
1522.81
1605.96
1689.83
1852.92
Relative Abundance
m/z

Figure S11

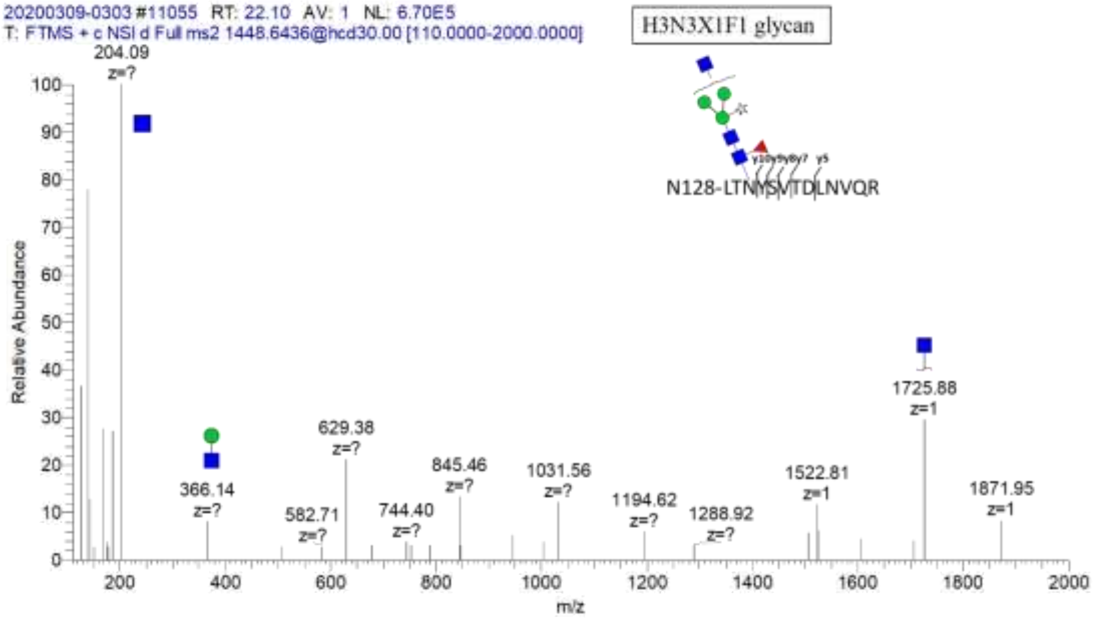

20200309-0303 #11055 RT: 22.10 AV: 1 NL: 6.70E5
T: FTMS + c NSI d Full ms2 1448.6436@hcd30.00 [110.0000-2000.0000]
H3N3X1F1 glycan
N128-LTNYSVTDLNVQR
204.09 z=?
366.14 z=?
582.71 z=?
629.38 z=?
744.40 z=?
845.46 z=?
1031.56 z=?
1194.62 z=?
1288.92 z=?
1522.81 z=1
1725.88 z=1
1871.95 z=1
Relative Abundance
m/z

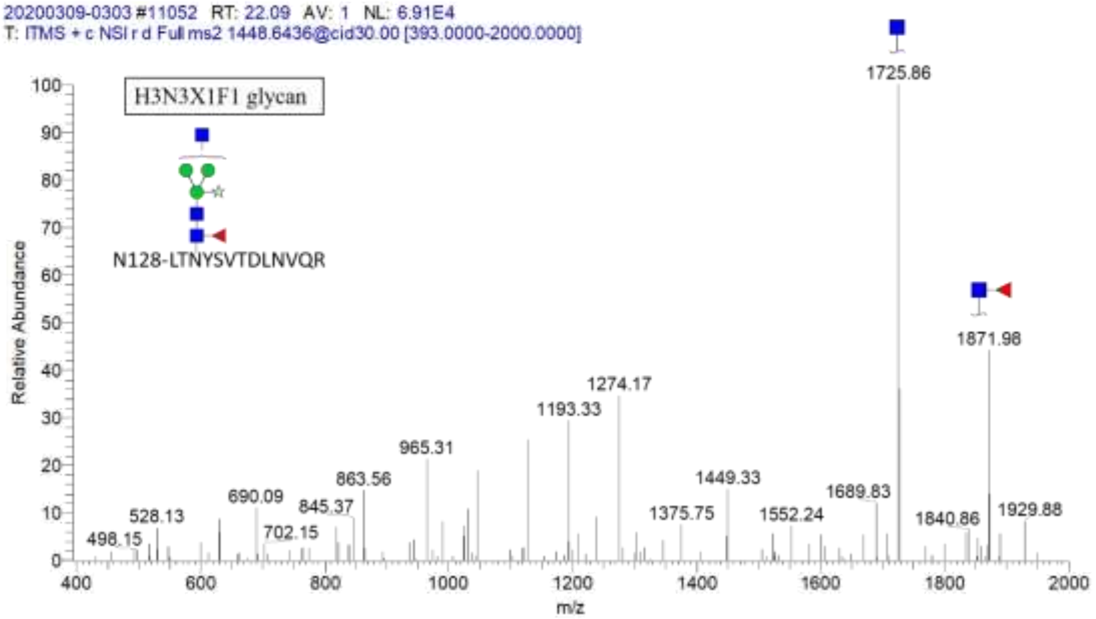

20200309-0303 #11052 RT: 22.09 AV: 1 NL: 6.91E4
T: ITMS + c NSI r d Full ms2 1448.6436@cid30.00 [393.0000-2000.0000]
H3N3X1F1 glycan
N128-LTNYSVTDLNVQR
1725.86
1871.98
1274.17
1193.33
965.31
863.56
1449.33
1689.83
690.09
845.37
1375.75
1552.24
1840.86
1929.88
528.13
702.15
498.15
Relative Abundance
m/z

Figure S12

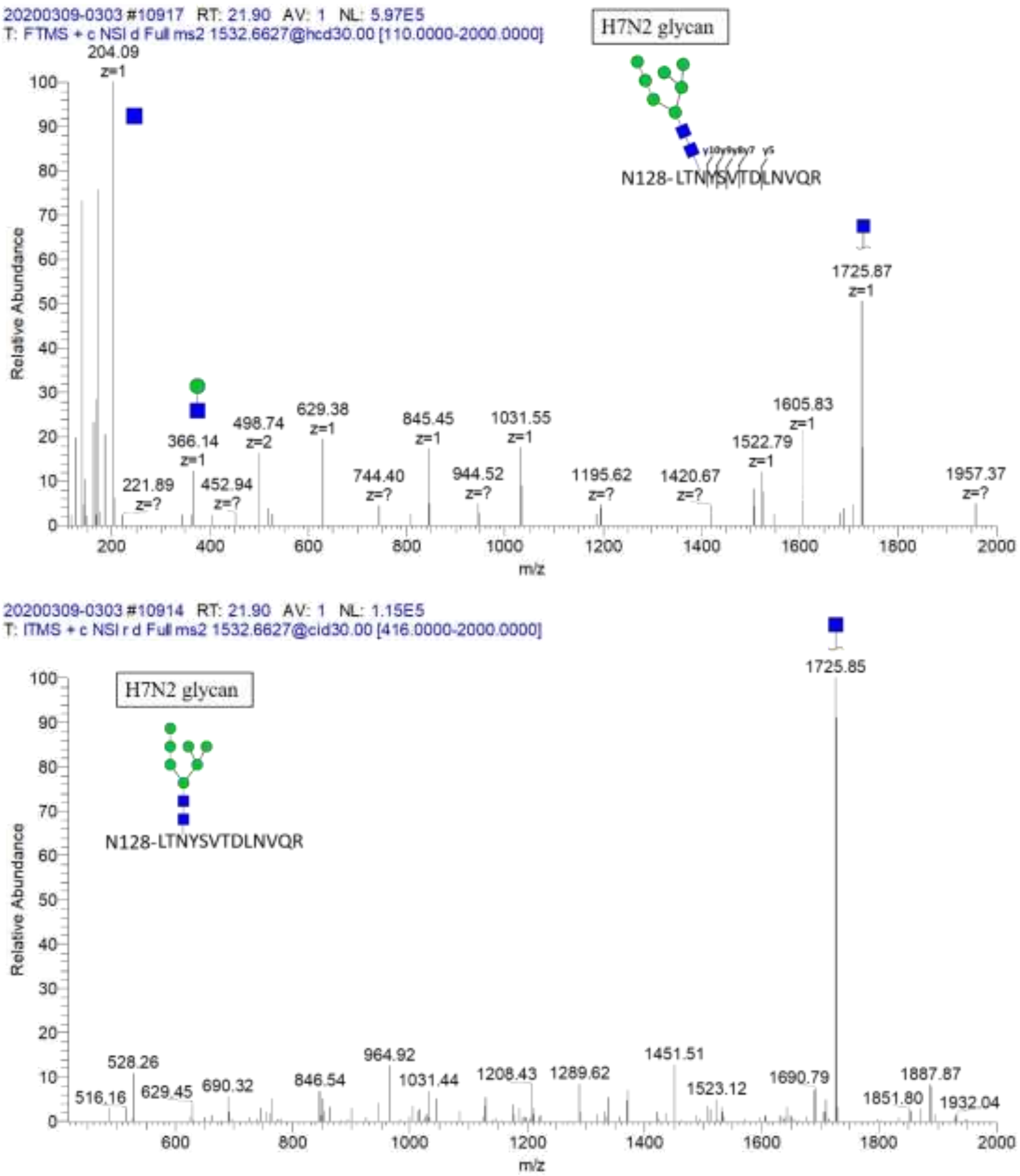
20200309-0303 #10917 RT: 21.90 AV: 1 NL: 5.97E5
T: FTMS + c NSI d Full ms2 1532.6627@hcd30.00 [110.0000-2000.0000]
H7N2 glycan
N128-LTNYSVTDLNVQR
Relative Abundance
m/z
204.09 z=1
366.14 z=1
221.89 z=?
452.94 z=?
498.74 z=2
629.38 z=1
744.40 z=?
845.45 z=1
944.52 z=?
1031.55 z=1
1195.62 z=?
1420.67 z=?
1522.79 z=1
1605.83 z=1
1725.87 z=1
1957.37 z=?
20200309-0303 #10914 RT: 21.90 AV: 1 NL: 1.15E5
T: ITMS + c NSI r d Full ms2 1532.6627@cid30.00 [416.0000-2000.0000]
H7N2 glycan
N128-LTNYSVTDLNVQR
Relative Abundance
m/z
1725.85
516.16
528.26
629.45
690.32
846.54
964.92
1031.44
1208.43
1289.62
1451.51
1523.12
1690.79
1851.80
1887.87
1932.04

Figure S13

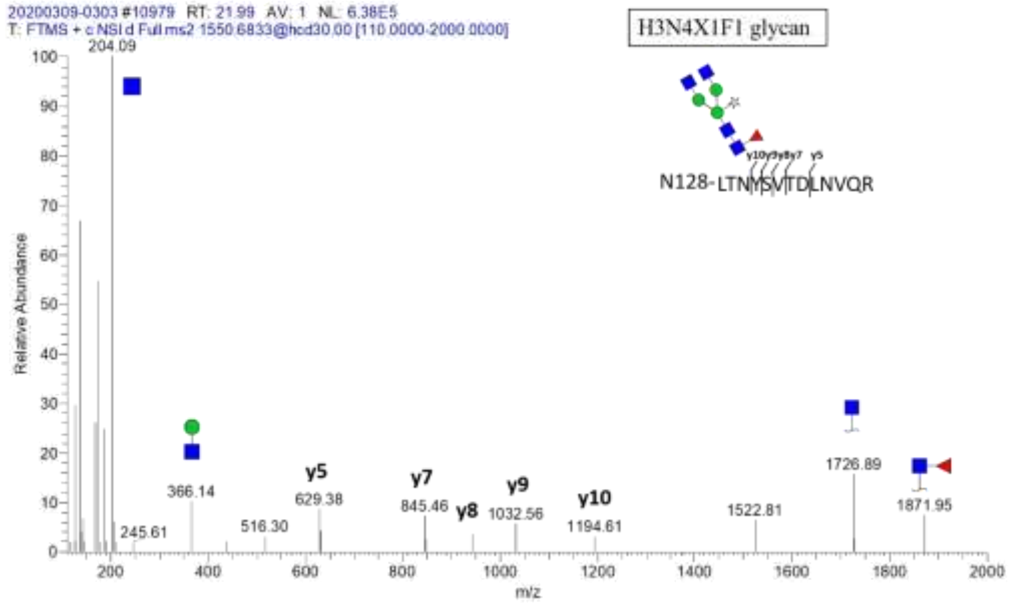
20200309-0303 #10979 RT: 21.99 AV: 1 NL: 6.38E5
T: FTMS + c NSI d Full ms2 1550.6833@hcd30.00 [110.0000-2000.0000]
H3N4X1F1 glycan
N128-LTNYSVTDLNVQR
Relative Abundance
m/z
204.09
366.14
245.61
516.30
y5
629.38
y7
845.46
y8
y9
1032.56
y10
1194.61
1522.81
1726.89
1871.95

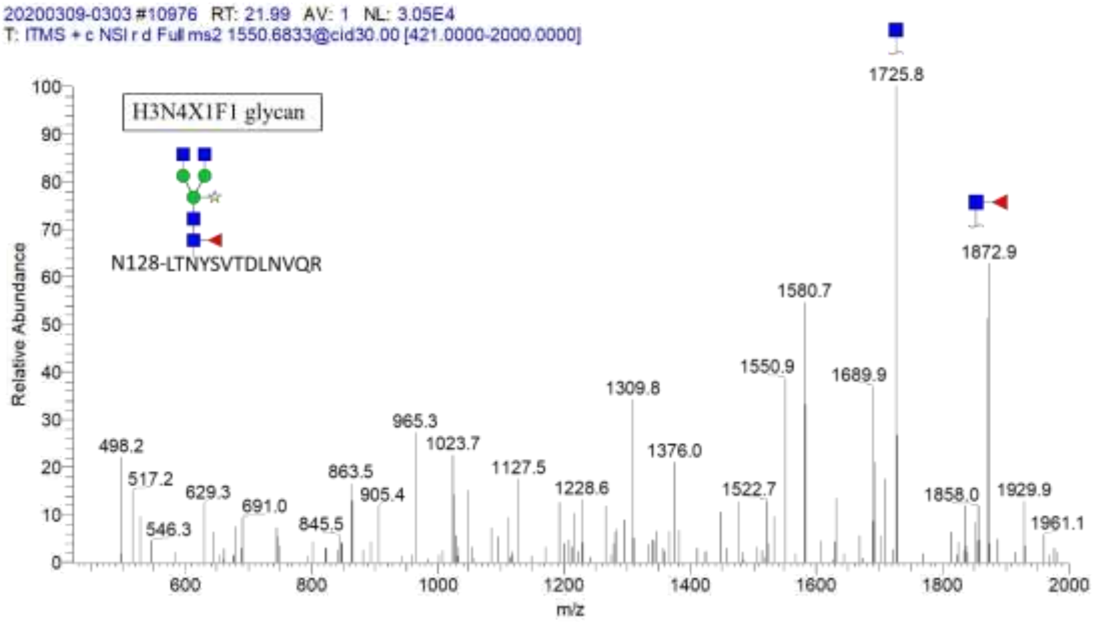
20200309-0303 #10976 RT: 21.99 AV: 1 NL: 3.05E4
T: ITMS + c NSI r d Full ms2 1550.6833@cid30.00 [421.0000-2000.0000]
H3N4X1F1 glycan
N128-LTNYSVTDLNVQR
Relative Abundance
m/z
1725.8
1872.9
1580.7
1550.9
1689.9
1309.8
965.3
1376.0
1023.7
498.2
517.2
629.3
691.0
546.3
863.5
905.4
845.5
1127.5
1228.6
1522.7
1858.0
1929.9
1961.1

Figure S14

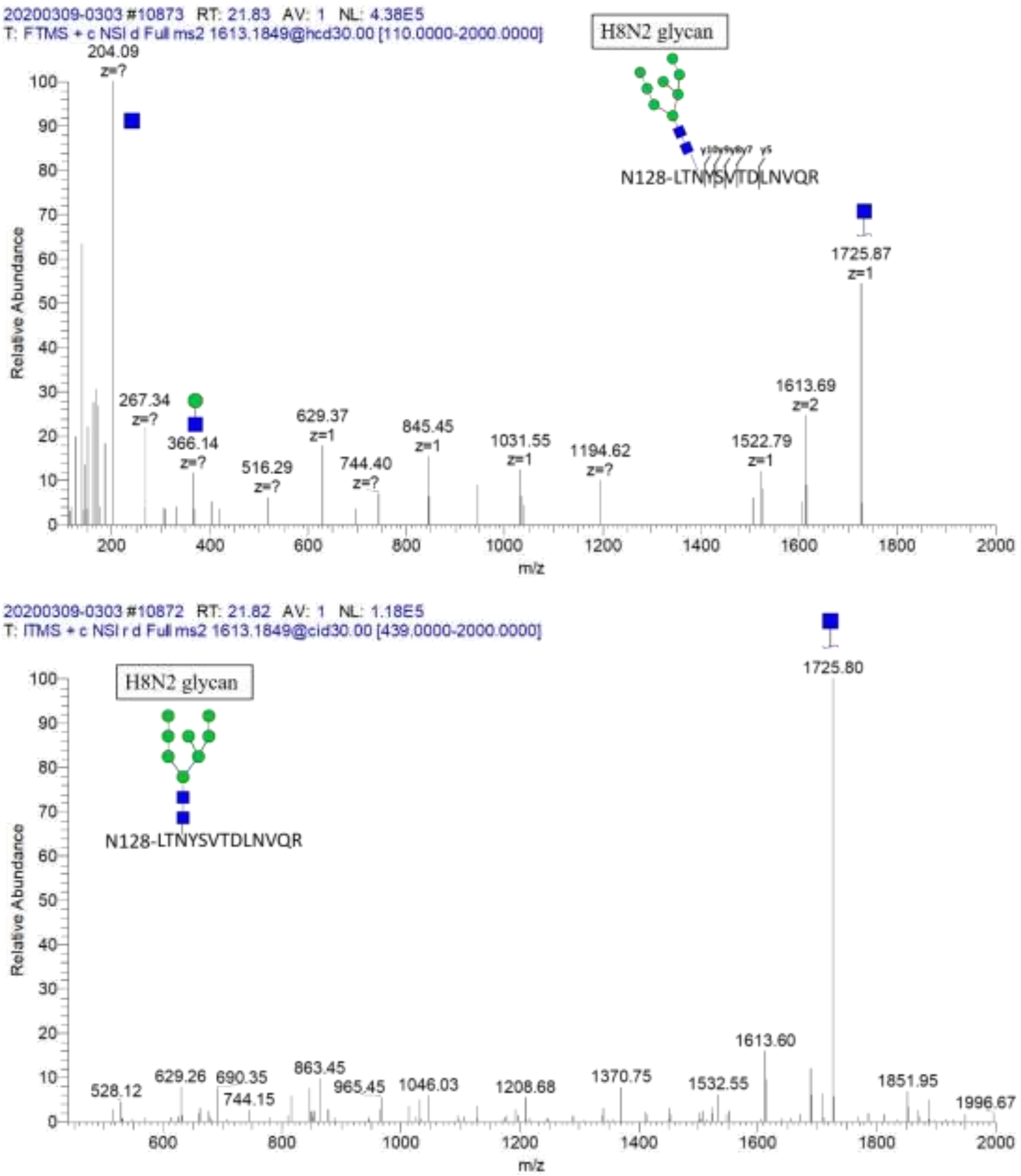

Supplementary Figures 5-14 | LC-MS/MS analysis of the glycopeptides and glycan generated from SS^{Ext}mIFNg(SP)$_{10}$. The assignment of each glyopeptides and glycan was based on the m/z values from both HCD and CID, as described in MATERIALS AND METHODS section.

A

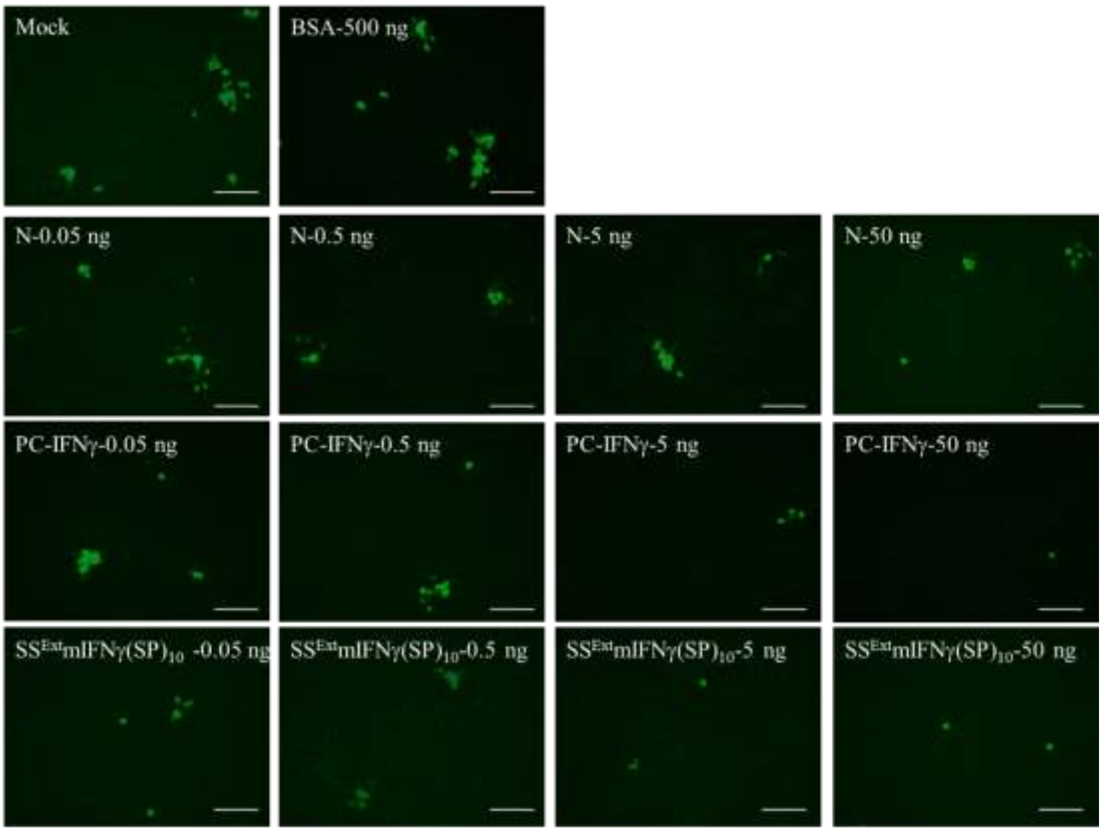
Mock
BSA-500 ng
N-0.05 ng
N-0.5 ng
N-5 ng
N-50 ng
PC-IFNγ-0.05 ng
PC-IFNγ-0.5 ng
PC-IFNγ-5 ng
PC-IFNγ-50 ng
SSExtmIFNγ(SP)10 -0.05 ng
SSExtmIFNγ(SP)10-0.5 ng
SSExtmIFNγ(SP)10-5 ng
SSExtmIFNγ(SP)10-50 ng

B

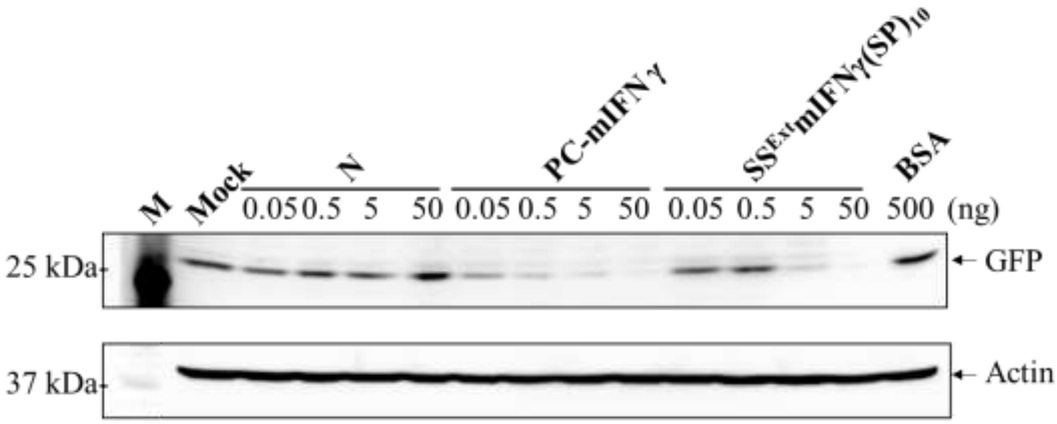
M
Mock
N
PC-mIFNγ
SSExtmIFNγ(SP)10
BSA
0.05 0.5 5 50 0.05 0.5 5 50 0.05 0.5 5 50 500 (ng)
25 kDa
GFP
37 kDa
Actin

Supplementary Figure 15 | Detection of $SS^{Ext}mIFN\gamma(SP)_{10}$ activity against SINV in SEK293-T cell. HEK293-T cells were pre-incubated with DMEM (Mock), 0.05, 0.5, 5, and 50 ng of protein extracts from *N. benthamiana* healthy leaves (N, the negative control), commercial IFNγ (PC-IFNγ, the positive control), $SS^{Ext}mIFN\gamma(SP)_{10}$, or 500 ng BSA for 12 h, followed by infection of SINV-eGFP at an M.O.I of 1.0. The eGFP signals were recorded by fluorescence microscopy **(A)** and accumulation level of eGFP and actin were analyzed by western blot of infected cell **(B)** at 24 hpi. Scale bar = 100 μm.
